# The human channel gating–modifying A749G *CACNA1D* (Cav1.3) variant induces a neurodevelopmental syndrome–like phenotype in mice

**DOI:** 10.1172/jci.insight.162100

**Published:** 2023-10-23

**Authors:** Nadine J. Ortner, Anupam Sah, Enrica Paradiso, Josef Shin, Strahinja Stojanovic, Niklas Hammer, Maria Haritonova, Nadja T. Hofer, Andrea Marcantoni, Laura Guarina, Petronel Tuluc, Tamara Theiner, Florian Pitterl, Karl Ebner, Herbert Oberacher, Emilio Carbone, Nadia Stefanova, Francesco Ferraguti, Nicolas Singewald, Jochen Roeper, Jörg Striessnig

**Affiliations:** 1 Department of Pharmacology and Toxicology, Institute of Pharmacy, Center for Molecular Biosciences Innsbruck, University of Innsbruck, Innsbruck, Austria.; 2 Department of Pharmacology, Medical University of Innsbruck, Innsbruck, Austria.; 3 Institute for Neurophysiology, Goethe University, Frankfurt, Germany.; 4 Department of Drug Science, N.I.S. Centre, University of Torino, Torino, Italy.; 5 Institute of Legal Medicine and Core Facility Metabolomics and; 6 Department of Neurology, Medical University of Innsbruck, Innsbruck, Austria.

**Keywords:** Neuroscience, Calcium channels, Calcium signaling, Mouse models

## Abstract

Germline de novo missense variants of the *CACNA1D* gene, encoding the pore-forming α1 subunit of Cav1.3 L-type Ca^2+^ channels (LTCCs), have been found in patients with neurodevelopmental and endocrine dysfunction, but their disease-causing potential is unproven. These variants alter channel gating, enabling enhanced Cav1.3 activity, suggesting Cav1.3 inhibition as a potential therapeutic option. Here we provide proof of the disease-causing nature of such gating-modifying *CACNA1D* variants using mice (Cav1.3^AG^) containing the A749G variant reported de novo in a patient with autism spectrum disorder (ASD) and intellectual impairment. In heterozygous mutants, native LTCC currents in adrenal chromaffin cells exhibited gating changes as predicted from heterologous expression. The A749G mutation induced aberrant excitability of dorsomedial striatum–projecting substantia nigra dopamine neurons and medium spiny neurons in the dorsal striatum. The phenotype observed in heterozygous mutants reproduced many of the abnormalities described within the human disease spectrum, including developmental delay, social deficit, and pronounced hyperactivity without major changes in gross neuroanatomy. Despite an approximately 7-fold higher sensitivity of A749G-containing channels to the LTCC inhibitor isradipine, oral pretreatment over 2 days did not rescue the hyperlocomotion. Cav1.3^AG^ mice confirm the pathogenicity of the A749G variant and point toward a pathogenetic role of altered signaling in the dopamine midbrain system.

## Introduction

*CACNA1D* encodes the pore-forming α1 subunit of the L-type Cav1.3 isoform of voltage-gated Ca^2+^ channels (1). Homozygous loss of Cav1.3 function in humans provided valuable insights into their physiological and pathophysiological roles, causing deafness and bradycardia without obvious neurological symptoms (2). Cav1.3^–/–^ mice resemble this phenotype (3), and their accessibility for more in-depth analyses revealed an impact of Cav1.3 deficiency on brain development (4–6), cellular excitability (7–11), and behavior (12–15). Although Cav1.3 channels only account for a minority of L-type Ca^2+^ channels (LTCCs) in the brain (16), their distinct negative voltage operation range enables them to specifically participate in the regulation of cellular excitability, synaptic morphology, or Ca^2+^-dependent gene expression (17–19). Given these important functions, it is not far fetched to assume that they might play a key role in the development of brain disorders. Indeed, in recent years, germline de novo missense variants of *CACNA1D* were identified in patients with neurodevelopmental disorders (for review, see ref. 20). So far, 9 *CACNA1D* germline variants in 12 affected individuals have been reported, but patients might be underdiagnosed since, until recently, *CACNA1D* has not been considered a high-risk gene for such conditions. The disease spectrum associated with these mutations comprises, to various degrees, autism spectrum disorder (ASD), seizures, autoaggression, muscle hypotonia, hyperactivity, primary aldosteronism, or hyperinsulinism (not all symptoms present in all patients; ref. 20) and is in line with the expression pattern and described physiological roles of Cav1.3 channels (for review, see ref. 17). However, to date, evidence for a disease-causing nature of these *CACNA1D* variants is only indirect. The mutated residues cluster around functionally important segments of the pore-forming Cav1.3 α1 protein, which together with auxiliary subunits precisely controls plasmalemmal Ca^2+^ influx in response to membrane depolarization. At rest, intracellular Ca^2+^ levels are ~10,000-fold lower compared with extracellular Ca^2+^ concentrations. Thus, tight control of Ca^2+^ influx is crucial to scale Ca^2+^ signaling events and prevent Ca^2+^ overload or toxicity. Functional studies in heterologous expression systems of these and similar somatic *CACNA1D* variants found in aldosterone-producing adenomas (APAs) revealed complex changes of channel gating, predicting increased Ca^2+^ influx especially at subthreshold potentials (20). An overall enhanced activity of Cav1.3 mutant channels is likely the disease-underlying mechanism because (a) mice (3) and humans (2) with hetero- and homozygous Cav1.3 deficiency lack a comparable phenotype; (b) disease-associated germline variants are absent in apparently healthy individuals (gnomAD database, https://gnomad.broadinstitute.org/); and (c) similar *CACNA1D* variants are associated with enhanced Ca^2+^ signaling and increased aldosterone secretion in APAs (20). Therefore, reducing the Ca^2+^ influx through Cav1.3 might represent a therapeutic option to alleviate symptoms in affected patients. Unfortunately, to date, no validated isoform-selective inhibitors of Cav1.3 exist (21–23). However, clinically approved and safe LTCC inhibitors, such as antihypertensives of the dihydropyridine (DHP) class, exist and could be readily repurposed for off-label treatment. However, it has to be considered that DHPs inhibit Cav1.2 LTCCs more potently (4), and applicable doses are limited due to Cav1.2-mediated cardiovascular side effects. Thus, identifying disease-underlying mechanisms and affected brain circuits may help to find alternative therapeutic targets that can be modulated with existing drugs.

Here, we aimed at establishing the first direct proof of the pathogenic nature of such gating-modifying *CACNA1D* variants by generating a mouse model containing one of the germline *CACNA1D* variants found in a patient with ASD and intellectual disability (A749G; Cav1.3^AG^ mouse line; ref. 24). Using this construct-valid model, we demonstrated that Cav1.3 channels with enhanced gating properties increased neuronal excitability in selective neural populations and resulted in a phenotype in adult mutant mice resembling many of the impairments observed in patients carrying such mutations. Furthermore, we identified the dopamine (DA) midbrain system as one key brain circuit affected by this *CACNA1D* channelopathy and established a multiple-dose oral administration regimen of the LTCC inhibitor isradipine resulting in therapeutically relevant plasma concentrations. However, short-term isradipine treatment in adult mice did not alleviate the hyperlocomotive phenotype.

## Results

### Cav1.3^AG^ mutant mice are viable, are fertile, and show a delayed gain of body weight without major endocrine dysfunctions.

We have introduced the p.A749G *CACNA1D* mutation (human nomenclature, corresponding to mutation A771G in mice) into C57BL/6N mice using CRISPR/Cas9 (Cav1.3^AG^ mouse line; Supplemental Figure 1, A–D; supplemental material available online with this article; https://doi.org/10.1172/jci.insight.162100DS1), which was found in a female patient diagnosed with ASD and intellectual disability (24) and caused typical changes of Cav1.3 channel gating associated with the spectrum of clinical abnormalities associated with the human disease (20, 25–27) (Supplemental Figure 1, E and F).

**Figure 1:**
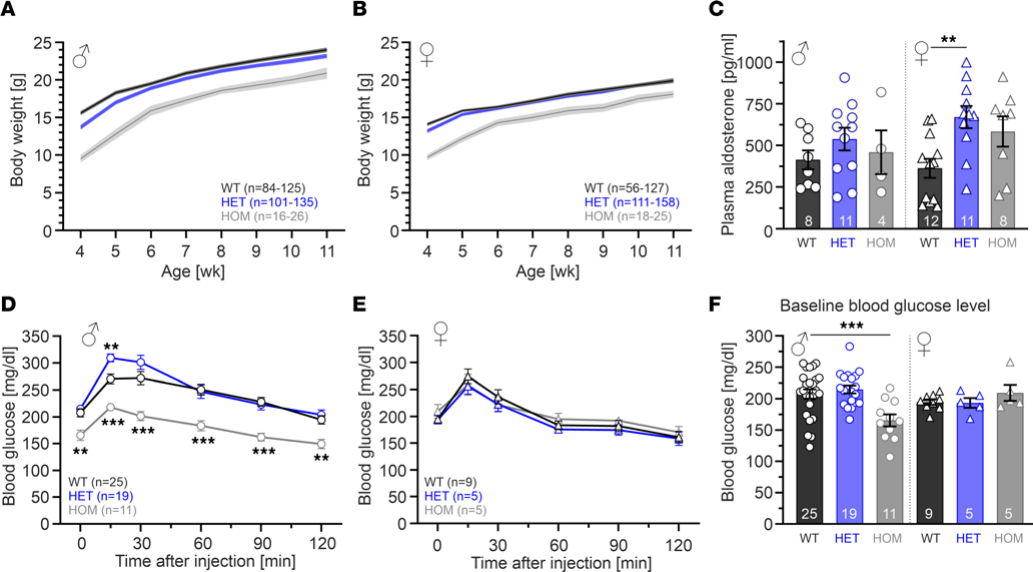
Delayed gain of body weight and no major endocrine dysfunctions in HET Cav1.3^AG^ mice. (**A** and **B**) Curves represent mean ± SEM (7-day bins) body weights of male (**A**) or female (**B**) WT, HET and HOM mice. For statistics at different ages, see Supplemental Figure 2, A and B. (**C**) Plasma aldosterone levels were similar in male WT and mutants (~15 wk) but significantly increased in female HETs compared with WT (~13 wk; 2-way ANOVA; genotype F_2,48_ = 5.06, *P* = 0.0101) with Šídák’s multiple-comparison test). (**D** and **E**) Blood glucose values of male (**D**; ~6 months) and female (**E**; ~13 wk) mice during an i.p. glucose test (1 mg/kg glucose i.p. injection after 6-hour fasting). Data are shown as mean ± SEM. Time point 0 represent the fasting basal blood glucose level before glucose injection. Mixed-effects model (**D**; males, time: F_3.8,193.2_ = 71.7, *P* < 0.001; genotype: F_2,52_ = 15.7, *P* < 0.001; interaction: F_10,252_ = 2.8; *P* = 0.0027) with Dunnett’s multiple-comparison post hoc test or 2-way ANOVA (**E**; females, time: F_5,95_ = 25.8, *P* < 0.001; no differences among genotypes). (**F**) Fasting basal blood glucose levels were significantly reduced in male HOMs only (2-way ANOVA; interaction F_2,68_ = 5.08, *P* = 0.0088) with Dunnett’s multiple-comparison test. ***P < 0.001, **P < 0.01.

Heterozygous (HET) and homozygous (HOM) Cav1.3^AG^ mutant mice were viable, and the presence of the mutant allele was confirmed by restriction digest and PCR approaches (Supplemental Figure 1, A and B). Cav1.3 α1 protein expression in whole-brain preparations was similar among genotypes (Supplemental Figure 1, G and H). Breeding of WT and HET mice resulted in a normal ratio of genotypes (WT, 54%; HET, 46%; *n* = 275), whereas in HET interbreedings, HOM mutants were slightly underrepresented (WT, 36%; HET, 49%; HOM, 15%; *n* = 371). No differences in sex distribution were observed. Mutant mice showed no obvious physical abnormalities, except for a reduced body weight that was already evident at birth. We monitored weekly weight gain in male and female mice starting after weaning (~4 weeks [wk]). At early developmental stages (~4–5 wk), HET and HOM mutants weighed significantly less (more pronounced for HOMs; Figure 1, A and B, and Supplemental Figure 2, A, and B). At adulthood (~11–12 wk), the body weight of HETs had caught up, in contrast to HOM mutants, which consistently weighed less, even at 1 year of age. The reduced body weight was not associated with changes of other morphometric parameters such as body length (Supplemental Figure 2C).

Since hyperaldosteronism and/or hypoglycemic hyperinsulinism were reported in some patients with mutations in *CACNA1D* (for review, see ref. 20), we studied the respective endocrine functions in our mouse model (Supplemental Methods). Plasma aldosterone levels of adult male mutant mice were similar to those of WT littermates, while in females, an increase by ~80% or ~60% in HETs or HOMs, respectively, was observed (statistically significant for HETs) (Figure 1C). We also monitored blood glucose levels at baseline (after 6 hours of fasting) and during an i.p. glucose tolerance test (Figure 1, D–F). Female mutants were indistinguishable from their WT littermates. In males, HOM mutants showed significantly lower blood glucose levels at baseline (in mg/dL: WT, 207.6 ± 7.5; HOM, 165.5 ± 9.7) and throughout the experiment.

In summary, the presence of 1 mutated allele in mice, analogous to a heterozygous patient, was sufficient to induce a developmental delay in body weight gain. Adult HETs show no major endocrine disturbances, except for a ~2-fold increase in aldosterone plasma levels in females. In contrast, HOM animals weighted significantly less throughout their lifespan, and male HOMs had significantly lower blood glucose levels, indicating a gene-dose dependency of the phenotype.

### Cav1.3^AG^ mutants exhibit context-dependent hyperlocomotion, aberrant grooming and rearing behavior, and social deficits.

Besides the reduced body weight, another obvious difference of Cav1.3^AG^ mutant mice was an increased locomotion in response to handling, which was again more pronounced in HOM animals. To systematically assess the behavioral phenotype, we performed a battery of behavioral tests with adult male WT, HET, and HOM mice. We first performed a home-cage activity measurement to assess baseline activity for 2 consecutive days. In general, baseline activity was reduced during the first half of the dark (active) phase in HOM mutants, while it did not differ among genotypes during the light (inactive) phase (Supplemental Figure 3A). Thus, behavioral tests were conducted during the light phase to prevent any potential confounding effects of locomotor activity.

**Figure 2:**
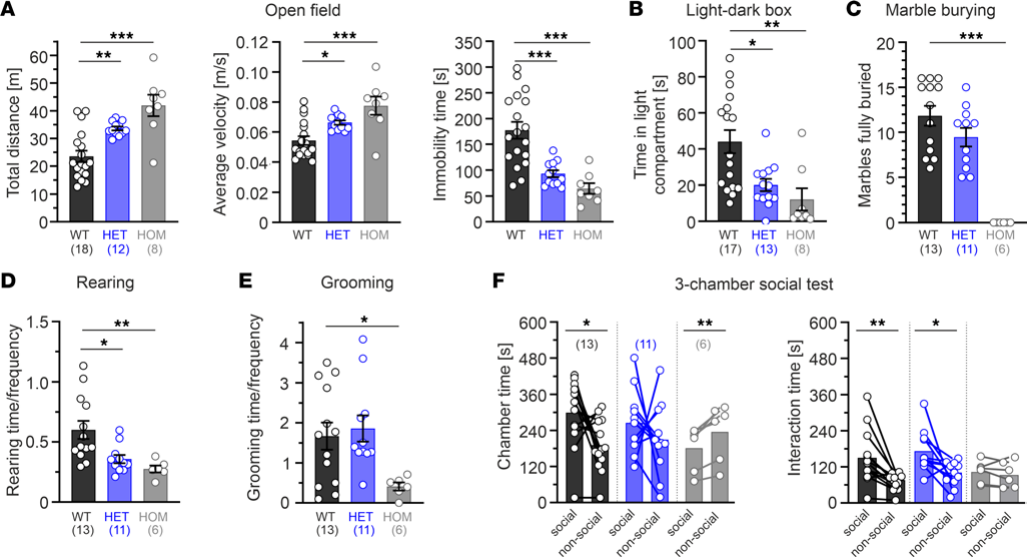
Cav1.3^AG^ mutant mice display increased locomotor activity and a social deficit. Data are shown as mean ± SEM. One-way ANOVA (**A** and **E**) or Kruskal-Wallis (**B**–**D**) with Dunnett’s or Dunn’s multiple-comparison post hoc test, respectively, were used, as was a paired Student’s *t* test (**F**). (**A**) In the open field (150 lux), compared with WT the distance traveled (left) and average speed (middle) was significantly increased in mutants, associated with less time spent immobile (right). Anxiety-related parameters (center/periphery time) were unchanged (Supplemental Figure 3E). (**B**) In the light-dark box (400 lux), mutant mice spent significantly less time in the light compartment. (**C**) HOM mutants did not bury any marble (out of 20) within a 30-minute time period. (**D** and **E**) Mice showed a significantly reduced ratio of rearing (**D**) or grooming (**E**) time over frequency in a novel environment in HET (rearing) and HOM (rearing and grooming) mice. A similar significant reduction for HOMs was observed in a familiar environment (data not shown). (**F**) Three-chamber social test. Quantification of the time spent in a chamber containing a grid with a unfamiliar mouse (“social”) or an empty grid (“nonsocial”) as well as direct nose-to-grid interaction time revealed a social deficit in HOM mutants. ***P < 0.001, **P < 0.01, *P < 0.05.

A gene-dose–dependent hyperlocomotion of mutant mice was reproducibly observed throughout all behavioral tests and was quantified for the open-field and elevated plus maze (EPM) tests. Both the total distance traveled (HET ~1.7-fold; HOM ~2.3-fold) as well as the average velocity (HET ~1.2-fold, HOM ~1.5-fold) were significantly higher in mutant mice, which also spent significantly less time immobile (decreased by HET ~50% or HOM ~70%; Figure 2A and Supplemental Figure 3, B–D). In both test settings, anxiety-related parameters — i.e., time spent in the anxiogenic center zone (open field) or open arm (EPM) — did not differ between mutant and WT mice (Supplemental Figure 3, E and F). In contrast, in the light-dark box, where a higher light intensity was used (400 lux compared with 100–150 lux in the other tests), HET and HOM mutant mice spent significantly less time in the lit compartment (decreased by ~50% and ~70%, respectively; Figure 2B).

Next, we focused on 2 main behavioral domains associated with ASD: repetitive/stereotypic behavior and sociability. A test associated with repetitive and obsessive-compulsive behavior is the marble burying test. While HET mice showed a comparable burying behavior to the WT animals (WT ~12, HET ~9 of 20 marbles), HOM mutants did not bury any marbles throughout the 30-minute test session (Figure 2C). Quantification of rearing and grooming behavior in a novel and familiar environment revealed similar time spent in rearing/grooming (Supplemental Figure 3, G and H), whereas the ratio of time over frequency was significantly reduced in HET (rearing) and HOM mutant mice (rearing + grooming; Figure 2, D and E; also observed for HOMs in the familiar environment, data not shown). To assess social behavior, we conducted the 3-chamber test with a “social” chamber containing an unfamiliar male mouse under a metal grid and a “nonsocial” chamber containing an identical empty metal grid. WT mice displayed the expected preference to spend more time in the social chamber compared with the nonsocial one (299 seconds [s] versus 191 s, respectively; *P* = 0.0170, paired Student’s *t* test, *n* = 13; Figure 2F, left panel). Conversely, no significant preference was found for HET mutants (266 s versus 210 s; *P* = 0.3774, *n* = 11), whereas HOM mice spent even more time in the nonsocial chamber (181 s versus 236 s; *P* = 0.0069, *n* = 6). Quantification of direct nose-to-grid interaction time in each chamber (Figure 2F, right panel) revealed a similar preference to interact more with the grid containing the unfamiliar mouse in WT and HETs (150 s versus 70 s [*P* = 0.0021] and 173 s versus 96 s [*P* = 0.0194], respectively). In contrast, HOM animals did not discriminate between the 2 grids (103 s versus 96 s; *P* = 0.4544).

In summary, Cav1.3^AG^ mutant mice displayed a gene-dose–dependent behavioral phenotype comprising enhanced locomotor activity upon exposure to a novel environment, aberrant grooming/rearing, enhanced anxiety-like behavior, and decreased sociability. Interestingly, no increased overall motor activity was observed in their home cage, indicating a context dependency of the locomotor phenotype.

### Gross brain morphology is unaltered in Cav1.3^AG^ mutant mice.

Since Cav1.3 channels participate in the regulation of cellular firing, spine morphology, and gene expression (for review, see ref. 17), and since altered volume and neuron number in certain brain regions have been reported in Cav1.3^–/–^ mice (4–6, 28), we examined whether a gain-of-function mutation induces neuroanatomical changes that could support or explain the observed behavioral phenotype in mutant mice.

**Figure 3:**
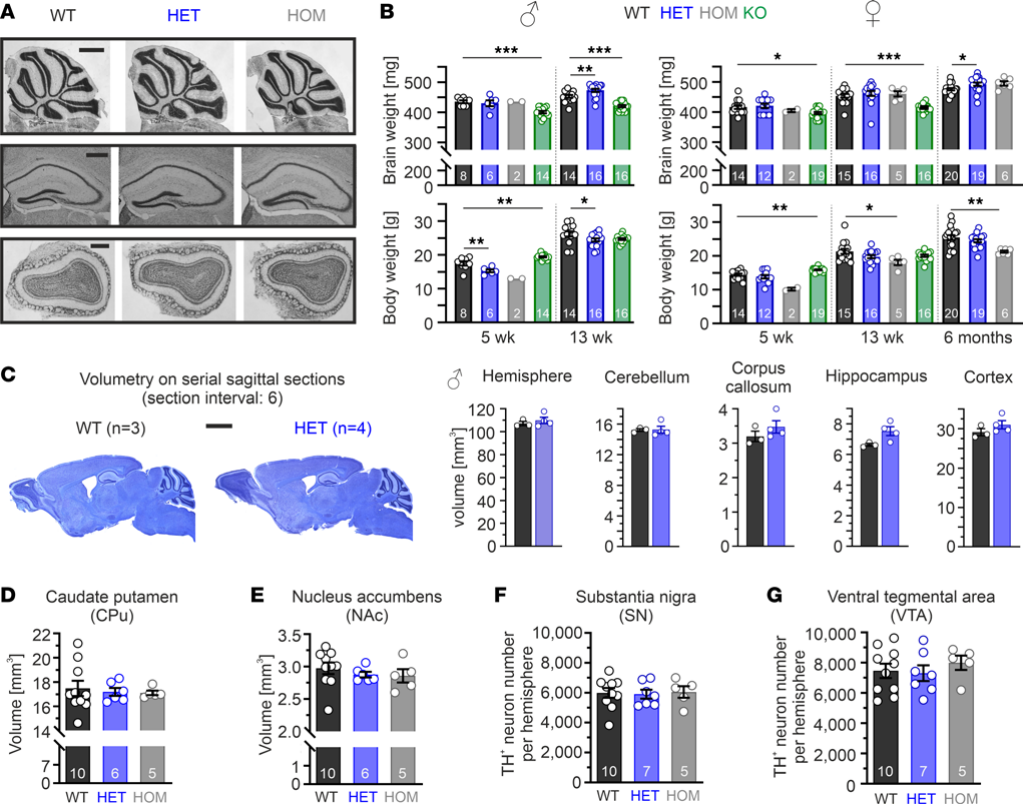
Similar brain morphology in Cav1.3^AG^ mutant mice. Data are shown as mean ± SEM. (**A**) Representative pictures of Nissl-stained brain sections from adult (13–15 wk) male mice of the cerebellum (*n* = 3–7/genotype), hippocampus (*n* = 5–10/genotype), and olfactory bulb (*n* = 5–7/genotype). Scale bars: 1 mm (cerebellum; top), 500 μm (hippocampus, middle; olfactory bulb, bottom). (**B**) Brain weight and respective body weight of male and female animals for the indicated age and number of animals. KO, Cav1.3-KO animals. Statistical analyses were performed with 1-way ANOVA with Dunnett’s multiple-comparison post hoc test. Due to the limited availability of homozygous mutants and respective experiments, we have excluded homozygous animals of the 5 wk cohort from statistical analysis due to the low *n*. (**C**) Left: Representative pictures of Nissl-stained sagittal brain sections from male WT and HET mice (olfactory bulb was not captures at the same level). Right: Comparable volumes of individual brain regions (Cavalieri principle; unpaired Student’s *t* test). Scale bar: 2 mm. (**D**–**G**) No statistically significant differences (1-way ANOVA) of the striatal volume (**D**, dorsal: caudate putamen [CPu]; **E**, ventral: nucleus accumbens [NAc]) or TH^+^ neuron number within the SN (**F**) and VTA (**G**) between adult male WT and mutant mice determined in serial TH-stained brain sections (Supplemental Figure 4, A and B). ***P < 0.001, **P < 0.01, *P < 0.05.

Qualitative and quantitative histologic assessment of Nissl-stained brain sections from adult male WT and mutant mice did not reveal any gross morphological abnormalities (Figure 3A). Interestingly, despite Cav1.3^AG^ mutant mice weighing less (Figure 3B, lower panel; Supplemental Figure 2, A and B), their brain weight was not reduced but showed a tendency to a slight increase with age (Figure 3B, upper panel). Volumetric measurements of whole hemispheres, cerebellum, corpus callosum, hippocampus, and cortex of HETs were comparable with those of WT (Figure 3C). This was in contrast to age-matched juvenile and adult Cav1.3-KO mice that possessed significantly smaller brains (reduced by ~7%; Figure 3B). Using male adult WT and mutant littermates, we further performed a more in-depth analysis of brain regions that highly express Cav1.3 (4, 9, 16, 29, 30) and are also associated with brain disorders such as ASD. In mutants, the total volume of the dorsal (caudate putamen [CPu]) and ventral striatum (nucleus accumbens [NAc]) was unchanged (Figure 3, D and E), and there were also no changes in volume along their rostro-caudal axis (Supplemental Figure 4C). Furthermore, mutants possessed comparable numbers of tyrosine-hydroxylase^+^ (TH^+^) DA midbrain neurons in the substantia nigra (SN) and ventral tegmental area (VTA) (unbiased stereology; Figure 3, F and G, and Supplemental Figure 4D). In the cerebellum, the density (cells/250 μm) and cell morphology of calbindin-positive Purkinje cells did not differ among genotypes (Supplemental Figure 5). Finally, in Ctip2/Satb2 or Ctip2/Tbr1 double-stained brain sections, the overall cortical architecture (layering) and number of stained cells showed no measurable alterations in HET or HOM mice (motor, sensory, infralimbic, and prelimbic cortex; Supplemental Figure 6).

Our results demonstrate that adult male mutant mice have similar brain weights and no major changes of the overall brain morphology, consistent with normal MRI scans from the majority of patients carrying such de novo *CACNA1D* missense variants (for review, see ref. 20).

### Gating changes of LTCC Ca^2+^ currents and altered firing in adrenal chromaffin cells from HET Cav1.3^AG^ mice.

**Figure 4:**
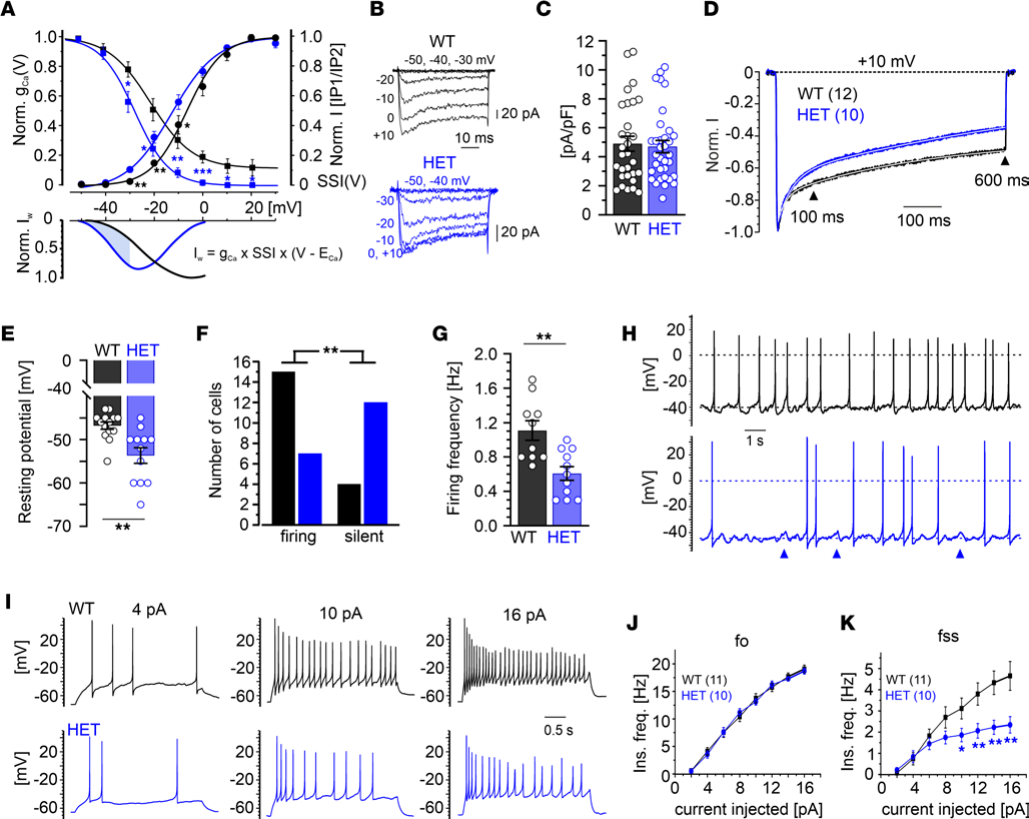
Gating changes and altered firing in acutely isolated MCCs from adult male HET Cav1.3^AG^ mice. (**A**) Voltage dependence of steady-state activation (normalized conductance-voltage curves; circles) and inactivation (squares) of WT (*n* = 7) and HET (*n* = 7) LTCC currents. Bottom, window Ca^2+^ current (I_w_) was increased for HET MCCs between −50 and −30 mV (shaded area). (**B**) Representative sets of pharmacologically isolated LTCC Ca^2+^ currents from a WT and HET MCC. (**C**) Similar current densities among genotypes (inward current at +10mV normalized to the cell size; WT, *n* = 30; HET, *n* = 34). (**D**) Averaged normalized LTCC currents during 600 ms depolarizations to +10 mV. Inactivation kinetics followed a double exponential time course: WT A_slow_ 38%, τ_slow_ 390 ms, A_fast_ 19%, τ_fast_ 18 ms, plateau (C) = 44%; HET A_slow_ 65%, τ_slow_ 904 ms, A_fast_ 31%, τ_fast_ 36 ms, C = 4%. (**E**) Compared with WT (*n* = 14), the resting membrane potential of HET MCCs (*n* = 12) was significantly decreased (*P* < 0.01, unpaired Student’s *t* test). (**F**) Percentage of spontaneously firing versus silent cells over a total of *n* = 19 WT or HET MCCs. Significance testing on categorical data was performed by RxC contingency tables and a χ^2^ test (*P* < 0.01). (**G**) Mean frequency of spontaneously firing HET MCCs (*n* = 11) was significantly decreased compared with WT (*n* = 10; *P* < 0.01, unpaired Student’s *t* test). (**H**) Current-clamp traces of representative WT and HET MCCs without current injection. Dashed lines indicate baseline; dotted line indicates 0 mV. Blue arrows indicate spontaneous subthreshold oscillations often observed in HET MCCs. (**I**) Representative AP traces at increasing current injections (4, 10, 16 pA) from a HP of −70 mV. (**J** and **K**) Plot of f_o_ (first interspike interval frequency) and f_ss_ (last interspike interval frequency) against the injected current. f_o_ is unaltered (**J**), while f_ss_ (**K**) exhibits a marked decrease with current injections above 10 pA in HET MCCs. Paired Student’s *t* test was used. **P < 0.01, *P < 0.05.

Since we detected no gross neuroanatomical changes, the behavioral phenotype might be primarily caused by altered brain function, present on the synaptic, cellular, and/or circuit level. Thus, we next studied potential effects on native Cav1.3 Ca^2+^ currents and electrical cell activity in Cav1.3^AG^ mice. For these experiments, we dissected spontaneously active adrenal mouse chromaffin cells (MCCs) (Figure 4), one of the few cell types in which Cav1.3 current components can be directly quantified (~25% of the total Ca^2+^ currents) and that rely on Cav1.3 to fine tune their excitability (10, 11, 31, 32).

First, in perforated patch-clamp recordings of primary cultured MCCs from adult male WT and HET mice, we tested whether the A749G mutation induces changes in Cav1.3 channel gating similar to those observed in heterologous expression systems (activation and inactivation shifted at more negative voltages and faster inactivation kinetics; refs. 25, 26) (Supplemental Figure 1, E and F, and Supplemental Table 1). In the absence of reliable Cav1.3-selective inhibitors, we studied mutation-induced effects on pharmacologically isolated LTCC currents (mediated by Cav1.2 and Cav1.3 to equal parts) (10). The voltage dependency of activation was significantly shifted by ~6 mV toward more negative membrane potentials in HET MCCs (voltage of half-maximal activation [V_0.5_] HET: –11.9 ± 1.2 mV; WT: –6.7 ± 0.3 mV; *n* = 7; *P* < 0.01; unpaired Student’s *t* test) (Figure 4, A and B). Normalization of the inward current at +10 mV to the cell size revealed similar maximal current densities (Figure 4C). To estimate the availability of LTCCs at various potentials, we examined the voltage-dependence of steady-state inactivation (SSI), which was also shifted by ~9 mV toward more negative potentials in HETs (voltage of half-maximal inactivation, V_0.5,inact_ WT: –18.4 ± 2.2; HET: –27.5 ± 1.9 mV; *n* = 7; *P* < 0.01; unpaired Student’s *t* test). Additionally, residual SSI at +20 mV was decreased from 13% ± 5% (WT) to 2% ± 2% (HET; *P* < 0.05; unpaired Student’s *t* test) (Figure 4A). The negative shift of the voltage dependence of channel gating predicts an increased constant background Ca^2+^ current (“window current” [I_W_]) in HETs at subthreshold potentials (Figure 4A, lower panel). Finally, we determined LTCC current inactivation kinetics during 600 ms pulses to +10 mV, which followed a double-exponential time course in both genotypes and was faster and more complete in HET MCCs (Figure 4D). This was also evident as a significantly increased percentage of inactivation at predefined time points (100 ms, WT [32% ± 3%, *n* = 12] and HET [41% ± 2%], *n* = 10,*P* < 0.05; 600 ms, WT [50% ± 3%, *n* = 12] and HET [67% ± 3%’, *n* = 10, *P* < 0.01; unpaired Student’s *t* test). These data confirm that native LTCC Ca^2+^ currents in HET mutant mice recapitulate the main mutation-induced gating changes observed in transfected cells (Supplemental Figure 1, E and F).

Given the critical role of Cav1.3 activity in regulating the MCC resting potential and the generation and frequency of spontaneous and evoked action potential (AP) firing (10, 11, 33), we studied whether these gating changes are sufficient to affect MCC firing. Indeed, the resting membrane potential (RMP) was, on average, more hyperpolarized by ~7 mV in HET MCCs compared with WT (Figure 4E). This may result from increased Ca^2+^ influx at subthreshold potentials due to the more pronounced I_w_ of mutant channels (Figure 4A), which consequently activates voltage-independent SK potassium channels with high-affinity for Ca^2+^. The hyperpolarization at rest was accompanied by a decreased number of spontaneously active MCCs from HET mutants (Figure 4F). In spontaneously firing cells, the AP frequency of HET MCCs was significantly lower with respect to WT (HET: 0.59 ± 0.08 Hz; WT: 1.09 ± 0.14 Hz) (Figure 4G). Interestingly, spontaneous subthreshold oscillations of 4–6 mV lasting 0.2–0.5 seconds were often observed in electrically silent and spontaneously active HET MCCs (blue arrows in Figure 4H), indicating the tendency of these cells to depolarize without reaching the threshold of AP firing (33). We also studied spike frequency adaptation during AP firing evoked from a HP of –70 mV (to silence spontaneously firing cells) by injections of current pulses of increasing amplitude (2–16 pA) (Figure 4, I–K). The firing frequency at the onset (f_o_) and steady-state (f_ss_) increased with current amplitude in WT and HET MCCs, suggesting spike frequency adaptation in both genotypes (Figure 4, J and K). There was no significant difference between f_o_ at any given current amplitude (Figure 4J), while at higher current amplitudes, f_ss_ was significantly lower by nearly 2-fold in HET compared with WT (Figure 4K). This suggests that MCCs adapt to a lower steady-state firing frequency in HET mutants, regardless of whether they fire spontaneously (Figure 4H) or are induced to fire by current injection (Figure 4I). Besides the above hypothesized effect via SK channels, this adaptation may also be, in part, the result of reduced Ca^2+^ entry during the long interspike intervals caused by the faster and more complete inactivation of Cav1.3^AG^ mutant channels.

Altogether, these data confirm that the heterozygous presence of A749G-containing Cav1.3 channels — i.e., ~25% of the total LTCC component — was sufficient to change native LTCC Ca^2+^ current properties and firing behavior in MCCs. This raises the question, if altered excitability also occurs in the brain and might contribute to the behavioral abnormalities observed in Cav1.3^AG^ mutant mice.

### DA midbrain and striatal medium spiny neurons (MSNs) show altered excitability in Cav1.3^AG^ HET mice.

Although Cav1.3 channels only account for ~10% of total LTCCs in the brain (16), their negative activation range compared with the major brain LTCC, Cav1.2, enables them to support pacemaker activity and regulate cellular excitability (9, 34). Cav1.3 is functionally expressed within the DA midbrain system, which is an important regulator of movement, motivation, reward-based learning, and cognitive functions. Thus, we studied 2 main cell populations within this brain circuit using in vitro brain slice electrophysiology. Given the robust hyperlocomotive phenotype of Cav1.3^AG^ mutant mice, we focused on the nigrostriatal pathway, where DA neurons in the SN project either to the dorsal medium striatum (DMS) or dorsal lateral striatum (DLS), where they release DA in an activity-dependent manner. In turn, striatal DA and its activation of D1 dopamine receptor (D1R) and D2R are crucial for control of locomotion; exploration of space, objects, and other mice; and learning and execution of action sequences (35, 36). Selective lesion experiments in striatal subregions demonstrated that D2R signaling in DMS but not DLS controls the level of exploratory activity (37). Thus, we expected enhanced activity in particular in DMS-projecting SN DA neurons in mutant mice.

**Figure 5:**
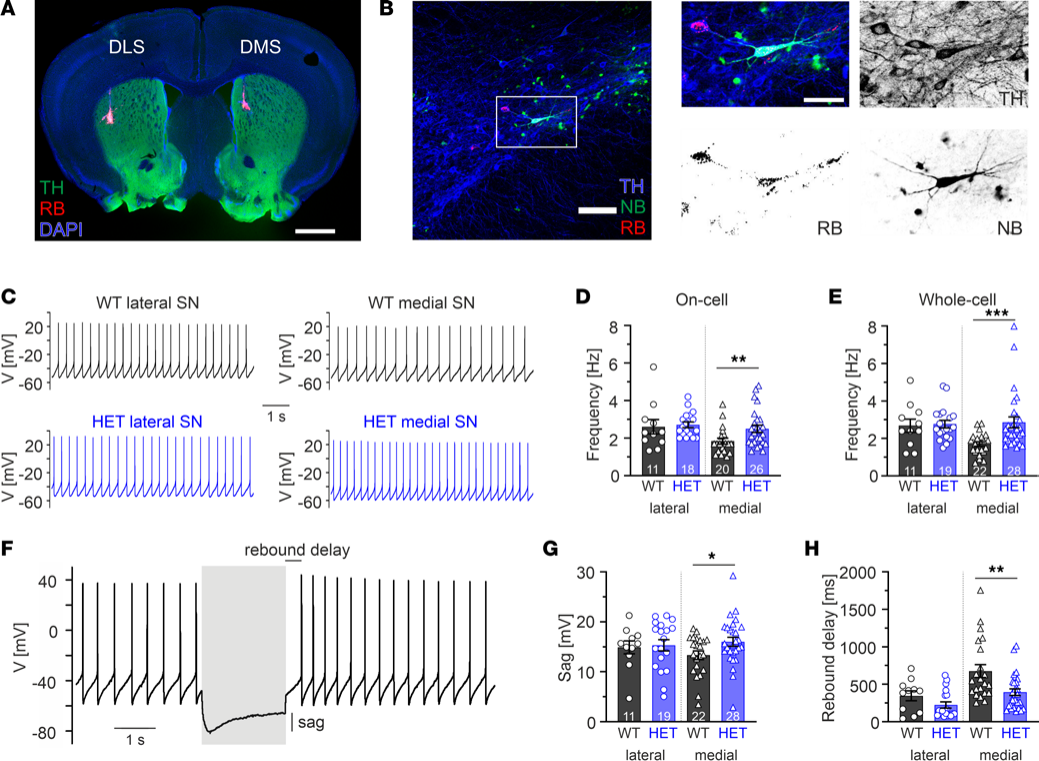
Projection-specific alterations of SN DA neuron firing in brain slices from HET Cav1.3^AG^ mice. Whole-cell patch-clamp recordings from DLS-projecting lSN (“lateral SN”) or DMS-projecting mSN DA neurons (“medial SN”) were performed in brain slices from adult male WT and HET mice. Data are shown as mean ± SEM. (**A**) Projection specificity was achieved by infusion of red beads into the DLS or DMS retrogradely labeling DLS- or DMS-projecting SN DA neurons (41). Scale bar: 1,000 μm. (**B**) Only DA cells (TH^+^; blue) that contained red beads (RB; red) and were filled with neurobiotin (NB; green) during the recording were included into the analysis. Scale bar: 100 μM, 50 μm (zoom). (**C**) Representative traces of autonomous pacemaking of both investigated SN DA neuron populations. DMS-projecting mSN DA neurons from HETs had a significantly higher firing frequency in the on-cell (**D**, *P* = 0.0069) and whole-cell configuration (**E**; *P* < 0.001) compared with WT (Mann-Whitney *U* test; Supplemental Table 2). (**F**) Upon hyperpolarization to approximately –80 mV (by 2-second current injection, gray rectangle), HET DMS-projecting mSN DA neurons showed a significantly increased sag component (**G**, *P* = 0.0395, unpaired Student’s *t* test) and faster rebound spiking (**H**; *P* = 0.0027, Mann-Whitney *U* test) (Supplemental Table 2). ***P < 0.001, **P < 0.01, *P < 0.05.

First, we performed current-clamp recordings in 2 SN DA cell populations from adult male WT and HET mice: TH^+^ DA neurons in the lateral SN (lSN) projecting to the DLS or in the medial SN (mSN) projecting to the DMS. The identification of specific striatal projection was achieved by retrograde labeling via infusion of red beads into either DLS or DMS (Figure 5, A and B). In vivo, SN DA neurons constantly fire APs in a tonic single spike or synaptically driven bursting mode (38, 39). In contrast, in ex vivo brain slices and in the absence of synaptic inputs, SN DA neurons showed spontaneous pacemaker firing. In accordance with our recent study (19), the mean discharge frequencies of DLS-projecting lSN DA neurons were faster compared with DMS-projecting mSN DA neurons (WT whole-cell mean frequency 2.7 ± 0.4 Hz versus 1.7 ± 0.1 Hz, respectively, *P* = 0.0029, unpaired Student’s *t* test) (Figure 5, C–E, and Supplemental Table 2). In contrast, in brain slices from HET animals, both SN DA neuron populations fired with similar mean frequencies, both in on-cell and whole-cell configuration (whole-cell HET 2.9 ± 0.3 Hz versus WT 2.8 ± 0.2 Hz), in the same range of WT DLS-projecting lSN neurons. These results demonstrate that DMS-projecting mSN DA neurons expressing mutant Cav1.3 channels displayed a projection-selective gain-of-function phenotype with elevated baseline firing (whole-cell HET 2.9 Hz versus WT 1.7 Hz, *P* < 0.001, Mann-Whitney *U* test), while the pacemaker rate of DLS-projecting lSN DA neurons was not altered by the presence of mutant channels (Figure 5, C–E, and Supplemental Table 2). We have recently shown that WT Cav1.3 channels amplify firing rates only in lSN but not mSN DA neurons (19), indicating that A749G Cav1.3 channels not only enhance a preexisting function of WT Cav1.3 channels in DMS-projecting mSN DA neurons but also, to our knowledge, establish a categorical novel gain of function in this DA subpopulation. In contrast, in DLS-projecting lSN DA neurons where Cav1.3 channels already accelerate pacemaking in WT neurons, the additional presence of mutant Cav1.3 channels did not further speed up pacing rates. We speculate that DLS-projecting lSN DA neurons are endowed with a selective homeostatic pacemaker plasticity to buffer the presence of mutant Cav1.3 channels, while DMS-projecting mSN DA neurons where WT Cav1.3 channels play no role in pacemaker control do not have that capacity and, thus, display a gain-of-function phenotype.

Mutant Cav1.3 channels might have not only direct electrogenic effects on firing rates but also affect expression of other relevant pacemaker ion channels, since LTCCs can participate in excitation-transcription coupling (17, 40) and, thus, may affect excitability through indirect mechanisms. To probe for this experimentally, we compared the response to 2-second hyperpolarizing current injections in both SN DA neuron populations from WTs and HETs, since this protocol reveals the function of hyperpolarization-activated cyclic nucleotide-gated (HCN) channels. We quantified the so-called sag component, a membrane depolarization indicating the activation of HCN channels in response to the membrane hyperpolarization to approximately –80 mV (Figure 5F, gray rectangle). While again no significant differences between WT and HET DLS-projecting lSN DA neurons were observed, DMS-projecting mSN DA neurons displayed a significantly larger sag component (WT 13.3 mV versus HET 16.0 mV, *P* = 0.0395, unpaired Student’s *t* test) associated with shorter rebound delay (WT 676.4 ms versus HET 394.7 ms, *P* = 0.0027, Mann-Whitney *U* test) (Figure 5, G and H, and Supplemental Table 2). These results indicate that mutant Cav1.3 channels selectively enhanced the function of other pacemaker channels in medial but not lateral DA SN neurons, and this might contribute to the accelerated gain-of-function pacemaker in this DA subpopulation. However, in vivo microdialysis measurements in the DMS of freely moving male WT and HET mice did not reveal differences in the extracellular DA levels in a familiar or novel environment (Supplemental Figure 7). Importantly, microdialysis did not reveal DA fluctuation on a faster time scale that might be driven by genotype-differences in electrical activity of DA neurons (Figure 5, C–E).

**Figure 6:**
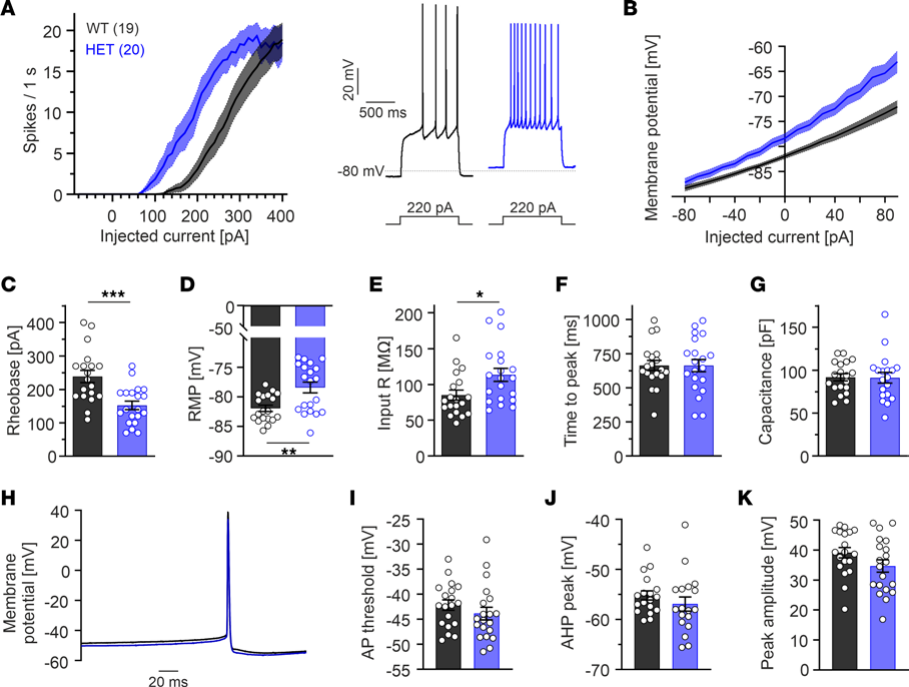
Striatal medium spiny neurons (MSNs) from HET Cav1.3^AG^ mice are hyperexcitable. Whole-cell patch-clamp recordings in acute brain slices from adult male and female WT and HET mice. Parameters of male and female mice did not differ significantly; therefore, data were pooled. Data are shown as mean ± SEM for the indicated number of cells. (**A**) Current-response curves (left, injected current versus number of elicited AP spikes) derived from 1-second current injections from –80 to 400 pA show that HET MSNs require less current stimulation to fire APs. Right, representative traces at 220 pA. (**B**) In MSNs from HETs, current injections elicited a stronger depolarization of the membrane potential compared with WT. (**C**–**G**) HET MSNs had a decreased rheobase current (current at which the first sweep with APs occurred, **C**; *P* < 0.001), more depolarized resting membrane potential (RMP, **D**; *P* = 0.0020), and increased input resistance in HETs (**E**; *P* = 0.0193) without changes of the time to peak measured at the rheobase (**F**) and cell size estimated by the capacitance (**G**). (**H**) Mean of the first AP at the rheobase sweep of WT (*n* = 19) and HET (*n* = 20) MSNs. (**I**–**K**) Manual analysis revealed no statistically significant differences for the AP threshold (**I**), afterhyperpolarization (AHP) peak (**J**), or AP peak amplitude (**K**). Statistical analyses were performed with unpaired Student’s *t* test. ***P < 0.001, *P < 0.05.

Next, we performed current-clamp recordings from the main striatal input target of SN DA neurons — i.e., GABAergic MSNs located in the dorsal striatum (41). MSNs are not spontaneously active, but in response to synaptic stimulation, they transition into an upstate that supports AP firing, which is associated with Cav1.3 activity (9, 42). To determine, if the A749G variant alters the intrinsic excitability of MSNs, we applied 1-second current injections from –80 to +400 pA (Figure 6A). The recordings were performed in acute brain slices from adult male and female WT and HET mice, and data were pooled, since no significant differences among sexes were observed. Plotting the injected current versus the elicited number of AP spikes (input-output curve) revealed a pronounced hyperexcitability of HET MSNs (Figure 6A). This means that lower somatic current injections were required to depolarize the membrane (Figure 6B) and to elicit AP firing (rheobase current: WT, 238.9 ± 18.4 pA; HET, 152.5 ± 12.7 pA) (Figure 6C). This was accompanied by a more depolarized RMP and significantly increased input resistance (WT, 85.1 ± 7.1 MΩ; HET, 113.5 ± 9.1 MΩ) (Figure 6, D and E). Neither the time to AP peak at the rheobase (Figure 6F), cell capacitance (Figure 6G), nor the AP shape (Figure 6, H–K) were affected. Moreover, we noted that HET MSNs more often displayed a depolarization block at high current injections (8 of 20) when compared with WT (2 of 19; Supplemental Figure 8).

Altogether, the heterozygous presence of the A749G *CACNA1D* variant induced a cell-autonomous projection-specific phenotype in the SN, with faster pacemaker activity, increased sag component and rebound spiking in DMS-projecting mSN DA neurons consistent with the observed hyperexploration phenotype of mutant mice. Moreover, their main input target, MSNs in the dorsal striatum, were hyperexcitable in HET mutants. These results indicate that the altered gating of A749G channels does change the functionality of basal ganglia neurons, thereby potentially driving the observed behavioral phenotypes.

### No clinically relevant rescue of the hyperlocomotive phenotype in Cav1.3^AG^ HET mice upon short-term oral in vivo isradipine administration.

**Figure 7:**
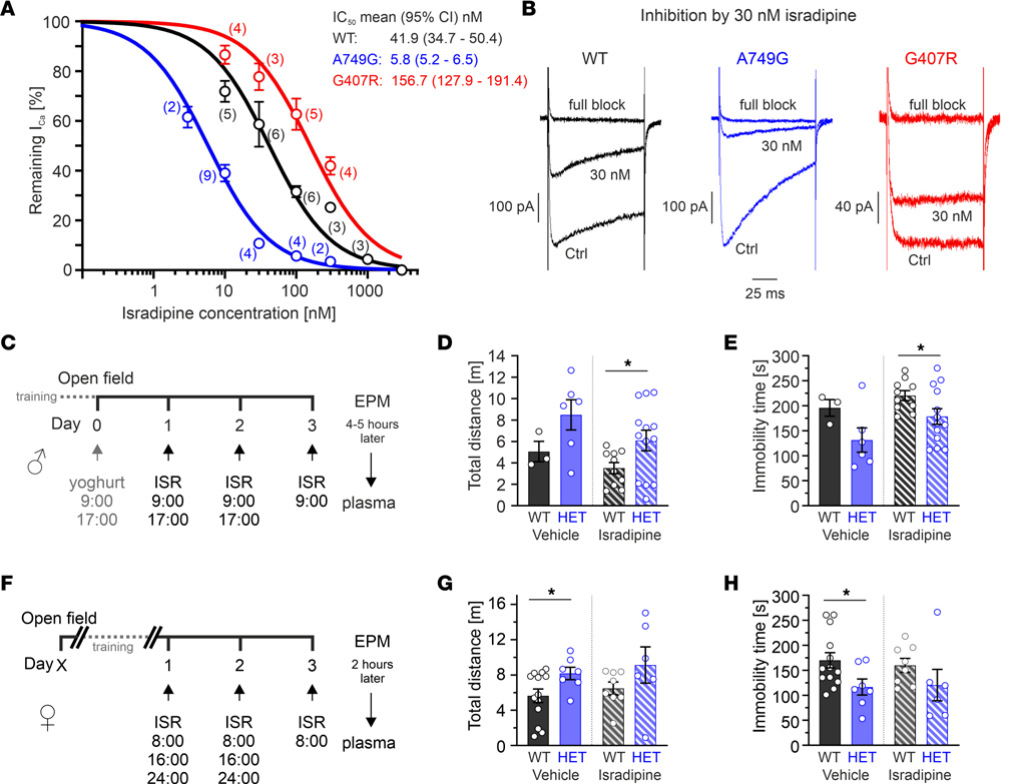
Variant-specific isradipine sensitivity in vitro and effects on locomotion by oral in vivo isradipine administration. (**A** and **B**) Human Cav1.3 α1-subunits were coexpressed with β3 and α2δ-1 in tsA-201 cells (HEK293 cells that stably express a SV40 temperature-sensitive T antigen) (15 mM Ca^2+^). Isradipine sensitivity was determined during 100 ms square pulses from a HP of –89.3 mV to V_max_ (0.1 Hz). Data are shown as mean ± SEM. (**A**) Concentration-response curves for the human C-terminally full-length Cav1.3 channel (hCav1.3_L_) WT, A749G, and G407R steady-state Ca^2+^ current (I_Ca_) inhibition by isradipine. IC_50_ values (means ± 95% CI) were obtained by fitting the curves using a Hill slope = 1 and top-bottom fixed (bottom = 0; top = 100). Statistical analyses were performed with extra sum-of-squares F test (*P* < 0.001). (**B**) Representative current traces for inhibition by 30 nM or 3 μM isradipine (full block). (**C** and **F**) Experimental design for the pharmacological rescue experiment in adult males (**C**) or females (**F**; 2 independent cohorts each). (**D** and **E**) Male mice received twice daily 0.5–1 mg isradipine (ISR) mixed into yogurt (1–2 mg/day; WT *n* = 10; HET *n* = 13). Three WT and 6 HET mice received yogurt only (vehicle group). On day 3, drug effects were tested in the EPM ~4–5 hours after the morning dose, and immediately afterward, plasma was isolated. Distance traveled (**D**) and immobility time (**E**) of vehicle- or isradipine-treated WT and HET animals. Statistical analyses were performed with unpaired Student’s *t* test (*P* = 0.041, **D**; *P* = 0.046, **E**). (**G** and **H**) Female mice received yogurt (vehicle group, WT *n* = 13; HET *n* = 7) or 0.1 mg ISR mixed into yogurt (0.3 mg/day; WT *n* = 8; HET *n* = 6) 3 times daily. On day 3, mice were tested in the EPM ~2 hours after the morning dose, and immediately afterward, plasma was taken. Distance traveled (**G**) and immobility time (**H**) of vehicle- or isradipine-treated WTs and HETs. Statistical analyses were performed with unpaired Student’s *t* test (*P* = 0.0447, **G**; *P* = 0.0389, **H**). Statistical analyses were performed with 2-way ANOVA; a significant comparison was only observed for the genotype (**D**, *P* = 0.0173; **E**, *P* = 0.0128; **G**, *P* = 0.0216; **H**, 0.0224). *P < 0.05.

Our data show that the A749G *CACNA1D* variant induced a behavioral phenotype in mice, without affecting gross neuroanatomy but by changing neuronal function. Attenuating these functional changes might help to alleviate associated symptoms in affected individuals. Since the underlying mechanism is likely an increase in activity of Cav1.3 mutant channels, reducing Ca^2+^ influx might represent a therapeutic option. In the absence of clinically available Cav1.3-selective blockers, we evaluated the activity of the nonselective DHP LTCC inhibitor isradipine (clinically approved antihypertensives) on transiently expressed human Cav1.3 WT and mutant channel complexes. The resulting concentration-response curves show a ~7-fold increased sensitivity of mutant A749G channels compared with WT (but a ~4-fold decreased sensitivity for G407R) (Figure 7, A and B), indicating the importance of testing the DHP sensitivity of Cav1.3 variants before considering off-label treatment with DHPs for respective patients.

The increased isradipine sensitivity of Cav1.3^AG^ channels in vitro prompted us to test if in vivo isradipine administration can attenuate the increased locomotion of HET Cav1.3^AG^ mice. This phenotype was selected because it was the most robust in HET mutants and is also burdensome when present in patients. We have established a multiple-dose oral treatment regimen where adult male mice were fed 0.5–1 mg isradipine twice a day over 3 consecutive days (extended-release formulation, mixed into fruit yogurt; Figure 7C). Before treatment onset, drug-naive mice were tested in the open field and HETs showed the expected hyperlocomotion (Supplemental Figure 9A). Following the treatment, drug effects were evaluated 4–5 hours after the last dose in the EPM, and plasma was taken immediately afterward. As an internal control to confirm the hyperlocomotion phenotype of HETs that was reproducibly observed in independent cohorts in the EPM (Supplemental Figure 3, B–D), we included a low *n* of vehicle–treated animals (yogurt only; WT *n* = 3, HET *n* = 6) and observed the expected ~2-fold increase in locomotion (Figure 7, D and E). We still observed a comparable difference in locomotion between the isradipine-treated WT and HETs, although absolute values of both locomotion parameters appeared to indicate slightly reduced locomotion for both genotypes (Figure 7, D and E). Since isradipine plasma concentrations at the time of testing varied over a concentration range from 2.4 to 72.6 ng/mL (Supplemental Figure 9B), we also tested the effect of isradipine exposure on locomotion (Figure 7, D and E). In WT mice, higher isradipine plasma levels correlated with a slight reduction of total distance traveled (Pearson correlation *r*^2^ 0.3322, *P* = 0.0392) and increased immobility time (Pearson correlation *r*^2^ 0.4110, *P* = 0.0182; Supplemental Figure 9, C and D). Although not significant, a similar trend toward reduced locomotion was found in isradipine-treated HETs. Since the obtained plasma levels were in a high supratherapeutic range, we refined the treatment schedule to achieve isradipine plasma levels within a range shown to be well tolerated in humans (up to 14.9–41.8 ng/mL) (43, 44). For these experiments, we used adult female mice and first confirmed the presence of the hyperlocomotion also in female mutants in the open field (Supplemental Figure 9E). Three daily doses of 0.1 mg isradipine mixed into yogurt every 8 hours on 3 consecutive days (Figure 7F) resulted in comparable mean plasma levels of ~15 or ~12 ng/mL in WT (*n* = 8) or HET females (*n* = 6), respectively, 2 hours after the last dose (Supplemental Figure 9F). Like in the open field, drug-naive HETs displayed increased motor activity (similar trend also in isradipine-treated HETs; Figure 7, G and H). There was no discernible effect of isradipine versus vehicle treatment on hyperlocomotion in WT or HET mice. In contrast to males, no correlation of plasma levels with locomotion was observed (Supplemental Figure 9, G and H).

Our data show that extended-release formulation of isradipine can be orally administered to mice, leading to effective plasma levels 2–5 hours after administration despite its short elimination half-life of < 7 minutes after i.v. injection (45). We found no evidence that this short-term administration of multiple doses of isradipine causes a meaningful reduction of context-dependent hyperlocomotion observed in our Cav1.3^AG^ disease model.

## Discussion

Imbalance of Ca^2+^ signaling caused by loss- or gain-of-function of various voltage-gated Ca^2+^ channels is associated with a wide range of human diseases (17, 46). In the present study, we provide the first direct evidence for the causative pathogenic nature of a human de novo *CACNA1D* (Cav1.3 α1) variant, A749G. We show that this point mutation is sufficient to induce a phenotype in mice similar to the corresponding human neurodevelopmental disease. As in the affected patients, the disease phenotype was already manifest in the heterozygous state indicative for a good transferability of findings from our disease mouse model to humans. This was also true for mice lacking Cav1.3 channels that reconstituted the bradycardic and deafness phenotype observed in humans (sinoatrial node dysfunction and deafness [SANDD] syndrome, OMIM #614896) (2, 3). Here, we focused on the A749G variant, identified in a female patient with ASD and intellectual disability, but without severe neurological (e.g., seizures) or endocrine features that might have made the interpretation of behavioral observations in our mouse model more complex. The pathogenic potential of mutations of the Cav1.3 A749 residue, which is highly conserved across all voltage-gated Ca^2+^ channel subtypes, is substantiated by missense variants for the corresponding residues in other voltage-gated Ca^2+^ channels. These analogous mutations are also associated with substantial disease burdens, like early infantile epileptic encephalopathy with developmental delay (Cav2.1 A711T, Cav2.3 A700T; refs. 47, 48) or childhood-onset cerebellar atrophy (Cav3.1 A961T; refs. 49, 50) (Supplemental Figure 1C). Interestingly, the Cav1.3 A749T mutation has also been found in 2 of the patients expressing *CACNA1D* variants (20, 51) inducing similar functional changes as A749G (unpublished data).

We and others have previously shown that the heterologously expressed A749G variant alters Cav1.3 channel gating, predicting enhanced activity in vivo (25–27). However, functionally characterized *CACNA1D* variants induce a complex phenotype combining both loss- and gain-of-function features in a variant-specific manner. A749G Cav1.3 channels require much weaker depolarizations to activate, but they also inactivate at more negative potentials and show faster inactivation kinetics. Our data in cultured MCCs (Figure 4) suggest that native Cav1.3 channels display the typical A749G gating changes. Cav1.3 mediates ~50% of the total isolated LTCC Ca^2+^ currents (mutant channels expected to contribute ~25%) that also show a shift of the voltage-dependency of channel gating by ~6–9 mV toward more hyperpolarized potentials and faster inactivation kinetics. Final proof that this is directly due to the aberrant gating of mutant channels will be addressed in future experiments in MCCs of double-mutant Cav1.3^AG^-Cav1.2DHP^–/–^ mice. These express Cav1.2 channels insensitive to DHPs, allowing selective inhibition of WT and mutant Cav1.3 channels (52). Similarly, we have previously confirmed typical gating changes of MCC LTCC currents — i.e., activation and inactivation at more negative potentials and slower inactivation kinetics, caused by the Timothy syndrome G406R Cav1.2 variant in TS2-neo mice (corresponds to the pathogenic G407R variant in human Cav1.3) (53). Current densities in MCCs were comparable in preparations from WT and mutant mice, suggesting that the mutation does not cause a major change in overall Cav1.3 current amplitudes.

Although not systematically evaluated, HET and HOM mutants showed no obvious difference in survival rate when bred for experiments up to 1 year. However, in interbreedings of HET animals, HOM mutants were underrepresented, and this trait was not associated with higher levels of early postnatal death. HET mutant mice showed a delayed gain of body weight, while HOM mutants weighed less throughout their life span. Since the reduced body weight was already evident at birth, it was most likely not due to reduced food intake and might be indicative of a global developmental delay. Despite lower body weight, no reduction in brain weight was observed. This was in accordance with clinical data from most human patients with *CACNA1D* mutations, where structural MRI did not reveal any gross morphological changes even in individuals with more severe symptoms (20). In contrast, our data of age-matched Cav1.3-KO mice revealed lower brain weights, consistent with reported reductions in volume and cell number in several brain areas (4–6, 28). For Cav1.3^AG^ mice, our gross neuroanatomical characterization does not exclude effects on other neuronal populations compared with those investigated here, nor does it exclude ultramicroscopic alterations (e.g., in pre- or postsynaptic structures that have been associated with neurological disorders).

Although the patient diagnosed with the A749G *CACNA1D* variant showed no endocrine dysfunctions, hyperaldosteronism and/or hyperinsulinism were reported in some individuals affected by high-risk *CACNA1D* variants. Cav1.3 channels are expressed in aldosterone-secreting zona glomerulosa cells of the adrenal gland and contribute to the Ca^2+^ signal driving aldosterone synthesis (54). Our data show that plasma aldosterone levels were slightly increased only in female mutants (significant in HETs). The absence of a more prominent hyperaldosteronism phenotype is in agreement with the lack of an endocrine phenotype in the patient diagnosed with the A749G *CACNA1D* variant (24). Interestingly, another *CACNA1D* mouse model, harboring the neighboring I750M variant, displays hyperaldosteronism and a much more severe neurological phenotype (55). This is also in agreement with human findings, since the individual affected by the I750M variant displayed a severe congenital phenotype characterized not only by hyperaldosteronism but also by seizures and other neurological abnormalities (56). Taken together, these 2 mouse models lead to phenotypes of different severity within the symptomatic spectrum observed in humans and may, therefore, be of value to study different phenotypic aspects of these *CACNA1D* channelopathies.

Cav1.3 channels are highly expressed in human insulin-secreting pancreatic β cells and appear to be involved in exocytosis (57), but their role in β cell function is less clear. Studies in 2 independent Cav1.3-KO mouse models reported diverging effects on glucose handling. While in one study, no major differences of Cav1.3 deficiency were observed on blood glucose and insulin levels upon fasting or a glucose challenge (3), another study found a hypoinsulinemic phenotype and impaired glucose tolerance (58). Our data from male HOM Cav1.3^AG^ mutants clearly indicate a role of Cav1.3 on blood glucose levels. They were significantly lower at baseline and during an i.p. glucose tolerance test and are, thus, compatible with the reported hyperinsulinemic hypoglycemia in 3 patients (56, 59, 60). Interestingly, we found no effects on glucose levels in female mutants, indicating a sex difference (e.g., also observed for female α2δ-1–KO mice that are less susceptible to diabetes; ref. 61). Together, these findings demonstrate that these 2 endocrine systems are indeed affected by the presence of the A749G mutation in a sex-specific manner but to a relatively minor extent, thus not causing overt disease in Cav1.3^AG^ mice.

Male HET mutants that correspond to the heterozygous disease state in humans showed no differences in body constitution (Supplemental Figure 2) or major endocrine features (Figure 1, C–F), excluding a confounding impact on the behavioral assessment. All behavioral abnormalities in Cav1.3^AG^ mice were more pronounced in HOMs, indicating a gene-dose dependency, as also observed for the body weight reduction. While indistinguishable in their home cages, upon handling mutant mice showed a strong escape response evident as enhanced locomotion and/or jumping, which would have even allowed blinded genotyping. When transferred into a test setting (e.g., novel open-field box), this hyperlocomotion was sustained throughout the experiment and was independent of lighting conditions (data not shown). Enhanced locomotion induced by handling or a novel environment (open field, EPM; Supplemental Figure 9E and Figure 7, G and H) was also present in female mice. The absence of hyperlocomotion in the home cage (constant environment, no handling/intervention) indicated a context dependency of this behavioral abnormality. Potential contributing factors include anxiety or sensory hypersensitivity. We did not observe an anxiety-like phenotype in the open field or EPM test, but we did in the light-dark box with very strong anxiogenic light exposure (400 lux), rendering a general anxiety phenotype unlikely. Cav1.3 channels are crucial for hearing, and Cav1.3 deficiency causes deafness in mice and humans (2, 3). Cav1.3^AG^ mice reacted to noise (e.g., clapping) — thus, they are not deaf — but we cannot exclude altered auditory sensitivity or processing. In line with ASD symptoms present in most patients expressing *CACNA1D* variants (and the major symptom in the A749G individual), we assessed behavioral domains that have been shown to be affected in ASD mouse models. HOM mutants are socially impaired; they lack the innate preference to spend more time with an unfamiliar mouse in the 3-chamber social test (Figure 2F). However, no stereotypies or repetitive behaviors were observed in HET and HOM mutants. We found that the ratio of rearing and grooming time/frequency was reduced, indicating that, while the rearing or grooming behavior was initiated, the highly specific and stereotyped pattern of sequential movement (also known as syntactic chain pattern) was abruptly terminated. Sequencing of behavior has been associated with the basal ganglia, including the striatum, as lesions within this brain area (in particular, the anterior dorsolateral striatum) can disrupt completion of normal grooming behavior (62). Additionally, the HOM mutant mice did not bury any marbles in the marble-burying test, reflecting impairments primarily in digging behavior, as also observed in other ASD animal models such as the C58/J (63) or a complete Shank3-KO model (64).

Although the aforementioned behaviors have been shown to be altered in several ASD mouse models (65, 66), like ASD itself, behavioral domains such as restricted/repetitive behaviors are also heterogeneous, ranging from “lower order” motor stereotypies to “higher order” cognitive behaviors such as insistence on sameness or rituals/routines (67, 68). Therefore, the normal behavior in a familiar, constant environment (home cage) on the one side and the context-dependent, induced motor hyperactivity as well as anxious behavior on the other side may also reflect an inflexibility to environmental changes.

Interestingly, several phenotypic aspects of Cav1.3^AG^ mice recapitulate observations in different hyperdopaminergic DA transporter (DAT) mouse models, such as mice deficient of DAT (KO; refs. 69–71) or expressing missense variants causing abnormal DA efflux (T356M identified in a patient with ASD; A559V associated with ADHD, ASD, and bipolar disorder; refs. 72–74). These include the strong escape reaction in response to handling (“darting” behavior; KO, A559V) as well as spontaneous hyperlocomotion in a novel environment (KO, T356M). Using cell type–specific silencing in selective striatal territories, Durieux and colleagues demonstrated that the control of locomotion in response to novelty is mediated by the DMS but not the DLS (37). In particular, they showed that diphteriatoxin-mediated ablation of D2R-expressing indirect pathway MSNs (iMSN) in the DMS resulted in novelty-induced hyperlocomotion. Since an increased DA tone in the DMS is also expected to inhibit iMSNs, the selective hyperexcitability of DMS-projecting mSN DA neurons detected in Cav1.3^AG^ mutants is an excellent candidate mechanism for novelty-induced hyperlocomotion. This DA phenotype is complemented by MSN hyperexcitability, which we also found in the dorsal striatum. A limitation of our study is that we did not distinguish the 2 main populations of striatal MSNs — i.e., D1R- and D2R-expressing MSNs. Although our data clearly show altered excitability of striatal neurons, more in-depth studies are needed to quantify the mutation-induced changes of excitability in defined MSN populations in the DMS. In addition, increased excitability of MSNs has been described in mouse models of ASD, such as Foxp1^+/–^ (D2R MSNs; ref. 75) and Shank3^–/–^ mice (D1R and D2R MSNs; ref. 76). We did not detect elevated extracellular DA levels within the DMS under basal conditions in the home cage or upon transfer into a novel environment (Supplemental Figure 7). Besides the possibility of different Cav1.3^AG^ effects on DA firing observed in vitro or in vivo, this could also reflect compensatory mechanisms limiting striatal DA release or increasing striatal DA reuptake. In addition, more sophisticated methods using genetically encoded DA sensors might be necessary to detect changes in fast DA fluctuations (77).

In contrast, MCCs displayed a decreased frequency of spontaneous and evoked AP firing (Figure 4). This observation shows that the effect of altered Cav1.3 channel gating does have different, context-dependent effects in distinct excitable cell populations (e.g., due to different firing patterns, channel expression, or different coupling between Cav1.3 and calcium-activated potassium channels) (19), which need to be studied in more detail in follow-up studies.

As described above, the in vivo consequences on Ca^2+^ influx in different cell populations are difficult to predict due to the complex gating changes induced by *CACNA1D* missense variants. The lack of a comparable phenotype upon heterozygous deficiency in mice and humans, however, points to an increase of Cav1.3 activity as the disease-underlying mechanism (at least in some neuron populations). Therefore, in the absence of reliable Cav1.3 selective inhibitors, we studied whether in vivo Cav1.3 inhibition by the DHP LTCC inhibitor isradipine could attenuate the locomotor phenotype in Cav1.3^AG^ mice. DHPs act preferentially on Cav1.2 LTCCs, and this limits the tolerable doses in humans due to the blood pressure–lowering action via arterial smooth muscle Cav1.2 inhibition. Efficient block of less-sensitive Cav1.3 channels (4, 78) would most likely require much higher doses than those approved for clinical application. In this context, our finding of a ~7-fold increased isradipine sensitivity of A749G-containing Cav1.3 LTCCs in vitro was encouraging (Figure 7A). Nevertheless, we first aimed for high, supratherapeutic isradipine plasma levels to ensure sufficient Cav1.3 target engagement. We have established, to our knowledge, the first oral, multiple-dose treatment regimen using an extended-release isradipine formulation resulting in plasma concentrations of ~28 ng/mL 4–5 hours after the last dose. These levels were tolerated by mice, as no mouse died and no abnormal behavior was observed in their home cages by blinded observers (2–4 daily inspections). We found that plasma levels correlated with reduced locomotion, evident as less distance traveled and higher immobility time (significant for WTs). However, we found no evidence that the isradipine treatment reduced hyperlocomotion in our Cav1.3^AG^ disease model beyond the plasma concentration–dependent reduction seen in male WT animals. To exclude the possibility that these supratherapeutic plasma levels induced confounding aversive effects, we adapted the isradipine administration to achieve peak plasma levels 2 hours after the last dose within a range well tolerated by humans in clinical studies (~13 ng/mL; Supplemental Figure 9F) (43, 44). Tolerability was excellent because all doses were voluntarily consumed within 5 minutes, and no adverse effects were recorded (e.g., abnormal appearance, diarrhea, loss of weight). No effect on locomotion in both WT and HET females was observed. Since isradipine efficiently passes the blood-brain-barrier with comparable concentrations in brain and peripheral tissue after acute application (45), we are confident that the achieved plasma levels (males, ~80 nM; females, ~40 nM) were sufficient to inhibit neuronal Cav1.3 (Cav1.3WT IC_50_ during SN DA neuron-like activity 7–17 nM; ref. 4), and in particular the more sensitive Cav1.3^AG^ mutant channels. Several factors may explain the lack of a rescue of the hyperlocomotion. Treatment onset at adult age could be too late — e.g., restricted time window for therapeutic intervention of distinct phenotypic features, as observed, for example, in Shank3-KO models (79, 80) or Kv7 K^+^ channel deficient mice (81). Another possibility is the requirement of longer, chronic drug exposures. Also, concomitant inhibition of Cav1.2 could affect the behavioral outcome. Finally, our observation of cell type–specific effects by the A749G variant raises the possibility that the complex gating changes may also induce a Cav1.3 loss of function in some cell populations, which would limit the feasibility of channel inhibition as a therapeutic strategy. This highlights the need for the identification of alternative drug targets, such as the DA midbrain system. Distinct symptoms may show different sensitivity to DHPs. This is indeed supported by findings in the I750M mouse model, in which a partial rescue was seen on motor symptoms in the rotarod test (55).

Taken together, our data provide proof of concept of the disease-causing nature of gating-modifying *CACNA1D* missense variants and show that the construct-valid Cav1.3^AG^ mouse model represents a suitable tool to study disease-underlying mechanisms and therapeutic strategies to improve clinical symptoms in affected patients.

## Methods

Supplemental Methods are available online with this article.

### Animals.

Adult male and female Cav1.3-KO mice (3) or Cav1.3^AG^ WT and mutant mice (expressing the human A749G *CACNA1D* variant) were used to study neuroanatomy, endocrine functions, behavior, endocrine and neuronal cell excitability (electrophysiology in cultured cells and brain slices), and the effect of oral administration of isradipine (Vascal uno 5 mg, extended-release formulation; Cheplapharm Arzneimittel GmbH). For a detailed description of the used methodology for all experiments, see Supplemental Methods.

### Statistics.

Data were analyzed using Clampfit 10.7 (Axon Instruments), SigmaPlot 12.5 (Systat Software), GraphPad Prism 9.0.1 (GraphPad Software), Origin 8.5 (OriginLab Corp.), Fitmaster (HEKA Electronics), and MATLAB. Values represent the mean ± SEM or mean and 95% CI for the number of experiments (*n*). Statistical testing was performed as indicated in the respective text, figure, and table legends; tests included paired or unpaired 2-tailed Student’s *t* test; Mann-Whitney *U* test; 1-way ANOVA with Dunnett’s multiple-comparison test; Kruskal-Wallis with Dunn’s multiple-comparison test; 2-way ANOVA with Dunnett’s or Šídák’s multiple-comparison test; mixed-effects model with Dunnett’s multiple-comparison test; RxC contingency tables and a χ^2^ test; and extra sum-of-squares F test. Statistical significance was set at *P* < 0.05. ***P < 0.001, **P < 0.01, *P < 0.05.

### Study approval.

Animal experiments were approved by the Austrian Animal Experimentation Ethics Board (BMWFW-66.008/0008-WF/II/3b/2014, BMWFW-66.008/0008-WF/V/3b/2017, BMWFW-66.008/0019-WF/V/3b/2017), by the University of Torino Animal Care and Use Committee (in accordance with the National Guide for the Care and Use of Laboratory Animals adopted by the Italian Ministry of Health; DGSAF 00 11710-P-26/07/2017), and by the German Regierungspräsidium Darmstadt (V54-19c20/15-F40/30).

### Data availability.

Values for all data points in graphs are reported in the Supporting Data Values file.

## Author contributions

NJO and J. Striessnig designed the study. NJO, EP, FF, N. Stefanova, PT, N. Singewald, EC, J. Striessnig, and JR designed research. NJO, TT, EP, PT, AS, MH, NTH, AM, LG, J. Shin, SS, NH, FP, HO, and KE performed research and analyzed data. NJO wrote the initial draft of the manuscript, with all authors contributing to the final version.

## Supplementary Material

Supplemental data

Supporting data values

## Version 1 09/12/2023

In-Press Preview

## Version 2 10/23/2023

Electronic publication
