# Supplementary material for: The human channel gating–modifying A749G CACNA1D (Cav1.3) variant induces a neurodevelopmental syndrome–like phenotype in mice
Source: JCI Insight. 2023 Oct 23;8(20):e162100. doi: 10.1172/jci.insight.162100 (PMC10619503; doi:10.1172/jci.insight.162100)
Supplement: Supplemental data [file jciinsight-8-162100-s151.pdf]

Supplemental methods

Animals

Animals were housed under a 12 hours light/dark cycle with food and water ad libitum. Cav1.3^{AG} mutant mice were generated using CRISPR/Cas9 (C57Bl/6N background; Taconic Biosciences). Male or female Cav1.3 knockout mice (KO, [1]), Cav1.3^{AG} wildtype (WT) and age-matched Cav1.3^{AG} mutant mice (heterozygous: HET; homozygous: HOM) were used (age and sex as indicated for respective experiments). Mice were genotyped either using an additional AvrII restriction site present in the mutant allele (inserted by a silent mutation, Supplemental Figure 1A) or a PCR approach with the following primers: CavAG_fwd: TACATTGCCACTTACAAAAACGGTGTTCA; CavAG_AG_fwd: CTGAATGTCTTCCTAGGCATTGCC; CavAG_WT_rev: GCCAAATTGTCCACAGCGATGGCCAA; CavAG_rev: TTAAAATTCCCAGCCAAGAGCTAATCAG (resulting in the following bands: WT: 974 bp + 600 bp; HET: 974 bp, 600 bp + 412 bp; HOM: 974 bp + 412 bp; The 974 bp band is barely visible, Supplemental Figure 1B).

Whole brain membrane preparations and Western Blots

For membrane preparations, brains were rapidly removed and immediately placed in ice-cold homogenization buffer containing 0.02 M $NaHCO_3$ and protease inhibitors (0.2 mM phenylmethylsulfonyl fluoride, 0.5 mM benzamidine and 2 mM iodoacetamide). Brains were homogenized by 10-20 strokes in a Dounce homogenizer, centrifuged (45,000 x g, 10 min, 4 °C) and washed three times (50 mM Tris-HCl, pH 7.4, containing the same protease inhibitor mix). Subsequently, membranes were resuspended in 1 ml of the same buffer and passed through a 25G and 27G cannula two times each. For separation of proteins, SDS-polyacrylamide gel electrophoresis (SDS page) was used. 33.4 µg of brain preparation were mixed with 6 µl 4X NuPAGE™ LDS Sample buffer (#NP0008, ThermoFisher) and incubated for 10 min at 70°C for denaturation. The mix was subsequently loaded together with a pre-stained molecular weight marker (PageRuler™; #26619, ThermoFisher) onto the SDS-page. The NuPAGE™ Tris-Acetate SDS running buffer was used (#LA0041, THermoFisher). Proteins were subsequently transferred onto a polyvinylidene fluoride membrane (PVDF, Immobilon-P Transfer membrane; Millipore, IPVH00010) at 20 V for 90 min in transfer buffer (NuPAGE™ Tris-Acetate SDS running buffer plus 20% MeOH). Blocking of the membranes was done for 2 h in blocking buffer (5% milkpowder in 20 mM Tris, 150 mM

NaCl, 0.5% Triton-X-100 and 0.1% Tween-20) and the primary antibody (rabbit anti-Cav1.3 α1: #ACC-005, Alomone labs; mouse anti-α-tubulin: CP06, Sigma Aldrich) was subsequently applied in blocking buffer over night at 4°C. After a washing step (5 x 5 min; 20 mM Tris, 150 mM NaCl, 0.5% Triton-X-100 and 0.1% Tween-20), the secondary antibody (goat anti-rabbit IgG-peroxidase; #A0545, Sigma-Aldrich; goat anti-mouse IgG HRP conjugate, 31430, Thermo Fisher Scientific) was applied in blocking buffer for 2 h at room temperature. After another washing step (5 x 5min), immunostained bands were visualized using SuperSignal™ West Femto Substrate (#34095, ThermoFischer) and a Fusion Fx7 Peqlab bioimager. Signal intensity was analyzed using ImageJ (NIH, https://imagej.net/ImageJ; [2]).

Intraperitoneal glucose tolerance test (IPGTT)

After 6 h fasting (water ad libitum, but no food or woodship beeding), basal fasting blood glucose levels of adult male (~24 wk) and female (~13 wk) Cav1.3^{AG} WT and mutant mice were determined via tail vein incision [3]. Subsequently, mice were injected i.p. with 1 mg/g glucose (in saline) and blood glucose levels were measured 15, 30, 60, 90 and 120 min after injection using a glucometer.

Plasma aldosterone measurements

To isolate plasma, mice were anesthetized with isoflurane, sacrificed by cervical dislocation and decapitated. Trunk blood was collected in EDTA-coated MiniCollect Tubes (Greiner-bio-one, Cat# 450531) and centrifuged for 10 min with 3000 x g at 4°C. The plasma was transferred to fresh Eppendorf tubes and stored at -80°C. Plasma aldosterone levels were measured using ELISA Kits (abcam, Cat# ab136933 or IBL, Cat# RE52301) according to the manufacturer's instructions.

Preparation of brain sections for Immunohistochemistry

All neuroanatomical analyses were performed with 40 µm thick coronal or sagittal brain sections from adult male animals. Brains from WT and mutant mice were processed in parallel and the experimenter was blinded to the genotype for all quantitative analyses. To assess the overall brain morphology (Figure 3A), brains were snapfrozen in -30 to -40°C cold 2-methylbutan and kept at -80°C until cutting using a

cryostat (Microm HM 560, Thermo Fisher Scientific) or perfused as described below. For all other analyses, mice were sacrificed with an overdose of Thiopental-Natrium (i.p.) and perfused through the heart (5 ml/min) using 0.9% saline (~3 min) and subsequently a fixative (~12 min) containing 4% PFA (and 15% of a saturated solution of picric acid) in phosphate buffer (PB). Brains were removed, stored at 4°C in 0.1 M PB until cutting (vibratome, VT1000S, Leica) and serial free-floating sections were stored in 0.1 M PB containing 0.05% NaN_3. Cryostat sections were post-fixed (4% PFA, 20min) before staining. Perfused vibratom-cut sections were mounted onto gelatin-coated glass slides and air-dried for at least 1 hour. For Nissl-staining, sections were dipped in Milli-Q water (1 min), followed by 5 min in 70% and then 100% ethanol, 9 min in 0.5% cresyl-violet solution, 2 min in 70% and then 100% ethanol and 1 min in Xylol until cover-slipped with Eukitt.

Volumetry of brain regions

The volume of different brain regions (cerebellum, corpus callosum, hippocampus and cortex) and the whole hemisphere was determined in sagittal Nissl-stained brain sections from 12 wk old male WT and HET mice (section interval: 6; analyzed sections per animal: 10; Cavalieri principle) using a Olympus BX51 Microscope equipped with a motorized stage and a computer-assisted image analysis system (Stereoinvestigator Software, Micro Bright Field Europe, Magdeburg, Germany).

Immunohistochemistry against Tyrosin-hydroxylase (TH) and Calbindin D-28k

After three washing steps in Tris-buffered saline (TBS), free-floating vibratome sections were incubated in blocking solution (20% normal goat serum and 0.3% Triton in TBS) for 1.5 h at room temperature (RT). Sections were subsequently exposed to the primary antibody (rabbit anti-TH: 1:4,000, Merck Millipore AB152; rabbit anti-calbindin D-28k: 1:20,000, Swant CB38) at 4°C for ~72 h, and after three times washing in TBS to the secondary biotinylated goat anti-rabbit antibody (1:500, Vector Laboratories BA-1000) at 4°C over night. The antibody solution contained 2% normal goat serum and 0.3% Triton in TBS. After washing (three times in TBS) sections were incubated in an avidin-biotin-peroxidase complex solution (Vectastain® ABC Kit, Vector Laboratories) for 1 h at RT. Following three washing steps in Tris-borate buffer (TB), sections were preincubated for 10 min at RT in 1 ml 3,3'diaminobenzidine

tetrahydrochloride (DAB, 0.5 mg/ml) and the immunoreaction was started by adding 0.003 % H_2O_2 for 2 min. The reaction was stopped by three washing steps in TBS. Finally, sections were mounted onto gelatin-coated glass slides, air-dried, dehydrated in graded alcohol (5 min in 50%, 70%, 95%, and 2x 100%), and 2x 10min in Xylol until cover-slipped with Eukitt.

Unbiased stereology of dopaminergic midbrain neurons

The number of TH^+ midbrain neurons in the substantia nigra (SN) or ventral tegmental area (VTA) were quantified in both hemispheres (Bregma -2.54 mm to -3.88 mm according to [4, 5]) by unbiased stereological analysis using the optical fractionator method with a Nikon E-800 or Olympus BX51 Microscope equipped with a motorized stage and a computer-assisted image analysis system (Stereoinvestigator Software, Micro Bright Field Europe, Magdeburg, Germany) and is reported as TH^+ neurons per hemisphere. The following counting parameters and settings were used: counting frame: 50 x 50 µm; grid size: 150 x 80 µm; section evaluation interval: 3; magnification: 2X and 100X (oil). Analyzed sections per animal: 9 (VTA) or 12 (SN). The Gundersen Coefficient errors (m=1) were ≤0.4.

Striatal volume measurements

The volume of the dorsal (caudate putamen; CPu) or ventral striatum (nucleus accumbens; NAc) in both hemispheres was determined according to the Cavalieri principle in TH-stained serial coronal sections with the same equipment as for the unbiased stereology (Bregma +1.94 mm to -1.94 mm according to The Mouse Brain in Stereotaxic Coordinates Third Edition [6]; section interval: 3; CPu 32 and NAc 11 sections/animal).

Cerebellar Purkinje cell density

The density of $calbindin^+$ Purkinje cells in cerebellar lobes 2, 3, 4/5, 6, 8, 9 and 10 was determined in sagittal sections (40 µm) taken around the midline, using the same equipment as for unbiased stereology. For the analysis, $calbindin^+$ cells were counted over a 250 µm long linear stretch within the same region per lobe in each animal and a mean number from 3-4 sections per animal was calculated.

Immunofluorescence staining for cortical analysis and analysis

For the analysis of the layering and neuron number within different cortical regions (sensory, S1; motor, M1; infralimbic, IL; prelimbic, PrL), two 40 µm thick sections per animal at Bregma level 1.94 to 1.78 mm (according to The Mouse Brain in Stereotaxic Coordinates Third Edition [6]) were double stained against Ctip2/Satb2 or Ctip2/Tbr1. Briefly, after 3 washing steps in TBS, free-floating sections were incubated in blocking solution (20% normal horse serum and 0.1% Triton in TBS) for 1 h at room temperature. Subsequently, sections were exposed to the primary antibody at 4°C for two days, and after 3 washing steps in TBS to the secondary antibodies at 4°C over night. The antibody solution contained 2% normal horse serum and 0.1% Triton in TBS. After three washing steps in TBS, sections were mounted onto gelatin-coated glass slides, cover-slipped with Vectashield and stored at 4°C in the dark until images were taken. Primary antibodies: rat monoclonal anti-Ctip2 (1:500, Abcam ab18465), rabbit monoclonal anti-Satb2 (1:500, Abcam ab92446), rabbit polyclonal anti-Tbr1 (1:1000, Abcam, ab31940). Secondary antibodies: donkey polyclonal anti-rat Cy3 (1:500, Jackson ImmunoResearch Europe Ltd 712-165-153), donkey anti-rabbit Alexa488 (1:1000, Invitrogen A-21206).

In each of the four investigated cortical regions, rectangles of 250 µm x 1666 µm (M1, S1) or 250 µm x 1146 µm (IL, PrL) covering all cortical layers were analyzed. The total number of $Ctip^{+}$, $Satb2^{+}$ or $Tbr1^{+}$ cells was determined using ImageJ (NIH, https://imagej.net/ImageJ; [2]). To visualize the distribution of stained cells throughout the different cortical layers, cell counts per lane were determined and plotted as mean ± SEM for each genotype (Supplemental Figure 6C-J).

tsA-201 cell culture and in vitro electrophysiology and pharmacology

tsA-201 cells (HEK293 cells that stably express a SV40 temperature-sensitive T-antigen, ECACC: 96121229) were cultured in Dulbecco's Modified Eagle's Medium (DMEM; Sigma-Aldrich, D6546) supplemented with 10% FBS (Gibco, 10270-106), 2 mM L-glutamine (Gibco, 25030-032), penicillin (10 U/ml; Sigma-Aldrich, P-3032) and streptomycin (10 µg/ml; Sigma-Aldrich, S-6501). Cells were kept in a humidified incubator (37°C, 5% CO_2) and split at ~80% confluency using 0.05% trypsin for cell dissociation (passage number did not exceed 20 passages). WT (Genebank accession number EU363339) or A749G and G407R mutant hCav1.3_L α1 subunits [7] were transiently expressed together

with β3 (rat, NM_012828), α2δ1 (rabbit, NM_001082276) and eGFP (transfection marker) using the Ca^{2+}-phosphate precipitation method as previously described [8]. The next day, cells were plated onto 35-mm culture dishes pre-coated with poly-L-lysine, kept at 30 °C and 5% CO_2, and all constructs were measured in parallel 48-72 h after transfection. Electrodes with a resistance of 1.5-3.5 MΩ were pulled from glass capillaries (Borosilicate glass, 64-0792, Harvard Apparatus, USA) using a micropipette puller (Sutter Instruments), and fire-polished with a MF-830 microforge (Narishige, Japan). tsA-201 cells were recorded in the whole-cell patch-clamp configuration using an Axopatch 200B amplifier (Axon Instruments, Foster City, CA) and were digitized (Digidata 1322A digitizer, Axon Instruments) at 50 kHz, low-pass filtered at 5 kHz and subsequently analyzed using the pClamp 10.2 software (Axon Instruments). Current leak subtraction was applied either online (P/4 subtraction; I-V protocol) or offline (5-s inactivation protocol; pharmacological experiments). Bath solution (in mM): 15 $CaCl_2$, 150 choline-Cl, 1 $MgCl_2$, 10 HEPES, adjusted to pH 7.3 with CsOH. Pipette solution (in mM): 135 CsCl, 10 Cs-EGTA, 1 $MgCl_2$, 10 HEPES, 4 ATP-Na_2 adjusted to pH 7.4 with CsOH. All voltages were corrected for a liquid junction potential of -9.3 mV [9].

Ca^{2+} current-voltage (I-V) relationships were obtained by applying a 20-ms long square pulse to various test potentials (Δ 5 mV) starting from a holding potential (HP) of -89.3 mV. Resulting I-V curves were fitted to the equation $I=G_{max}(V-V_{rev})/(1+exp[-(V-V_{0.5})/k])$ where I is the peak current amplitude, G_{max} is the maximum conductance, V is the test potential, V_{rev} is the extrapolated reversal potential, $V_{0.5}$ is the voltage of half-maximal activation and k is the slope factor. The conductance (G) was calculated using $G=(-I*1000)/(V_{rev}-V)$. The inactivation time course of WT und mutant Cav1.3 channels was determined during a 5-s long depolarizing pulse to V_{max} (voltage of maximal activation). Drug effects were measured with a 100ms long square pulse from the HP of -89.3 mV to V_{max} (0.1 Hz). Cells were superfused using an air pressure-driven perfusion system (BPS-8 Valve Control System, ALA Scientific Instruments) with bath solution in the presence or absence of isradipine (ISR; Fisher scientific, 50-850-70001) and a flow rate of ~0.5 ml/min. Respective ISR stocks were prepared in DMSO and freshly diluted 1:1000 in bath solution to the final concentration prior to the experiment. Drug/vehicle application was started after at least three constant control sweeps during perfusion with bath solution. On each recording day, individual control recordings with vehicle only were performed using the same tubes subsequently used for ISR experiments to exclude the possibility of contamination. Drug effects were corrected for linear current decay ("run-down") measured in these control cells. A part of the WT data has been published

in [10] together with the S652L mutant (that was measured partly in parallel with the here reported A749G and G407R variants) and was reanalyzed for the current study.

Cell culture and electrophysiological recordings of adrenal mouse chromaffin cells (MCCs)

Chromaffin cells were obtained from male 2 months old Cav1.3^{AG} WT and HET mice. Under sterile conditions the abdomen was opened, the adrenal glands were isolated, and transferred to an ice cold Ca^{2+} and Mg^{2+} free Locke's buffer containing (in mM) 154 NaCl, 3.6 KCl, 5.6 $NaHCO_3$, 5.6 glucose and 10 HEPES, pH 7.4 [11, 12]. Under a dissecting microscope the adrenal glands were decapsulated and subsequently subjected to an enzymatic dissociation with 20-25 units/ml papain (Worthington Biochemical Corporation, Segrate, Italy) dissolved in DMEM (GIBCO, Invitrogen Life Technologies, Monza, Italy) supplemented with 1.5 mM of L-cysteine, 1 mM of $CaCl_2$ and 0.5 mM of EDTA (Sigma Aldrich, Munich, Germany) for 25-30 minutes at 37°C in a water saturated atmosphere with 5% CO_2. Afterwards, two washing steps were performed with DMEM supplemented with 1 mM $CaCl_2$ and 10 mg/ml of BSA (Sigma Aldrich). Adrenal medullas were re-suspended in DMEM containing 1% pen/strep and 15% fetal bovine serum (both from Sigma Aldrich) and were mechanically dissociated with a fire polished Pasteur pipette. A drop (100 µL) of this concentrated cell suspension was plated on poly-ornithine (1mg/ml) and laminin (5 µg/ml) coated Petri dishes and subsequently (30 minutes later) 1.9 ml of DMEM containing 1% pen/strep and 15% fetal bovine serum (all from Sigma Aldrich) was added. The primary chromaffin cell cultures were kept in an incubator at 37°C at water saturated atmosphere with 5% CO_2. Measurements were performed on cultured MCCs two to five days after plating.

Macroscopic whole-cell Ca^{2+} currents and action potentials (APs) were recorded in perforated-patch conditions using a multiclamp 700-B amplifier and pClamp 10.0 software (Molecular Devices, Sunnyvale, CA, USA) [12, 13]. Traces were sampled at 10 KHz using a Digidata 1440A acquisition interface (Molecular Devices, Sunnyvale, CA, USA) and filtered using a low-pass Bessel filter set at 1-2 KHz. Long Ca^{2+} current traces (600 ms) used to determine the time course of current inactivation were filtered at 400 Hz [14]. Borosilicate glass pipettes (Kimble Chase life science, Vineland, NJ, USA) with a resistance of 2-3 MΩ were dipped in an Eppendorf tube containing intracellular solution before being back filled with the same solution containing 500 µg/ml of amphotericin B (Sigma Aldrich, Munich, Germany), dissolved in DMSO (Sigma Aldrich, Munich, Germany) [15]. Recordings started after

amphotericin B lowered the access resistance below 15 MΩ (5-10 min). Series resistance was compensated by 60-80% and monitored throughout the experiment. Fast capacitive transients during step depolarizations (in voltage-clamp mode) were minimized online by the use of the patch clamp analogue compensation. Uncompensated capacitive currents were further reduced by subtracting the averaged currents in response to P/4 hyperpolarizing pulses. Off-line data analysis was performed with pClamp 10.0 software.

Protocols: The steady-state activation was determined by 50 ms long step depolarizations starting from a holding potential (HP) of -70 mV. Steady-state inactivation was calculated as the ratio of Ca^{2+} currents during 30 ms long test pulses to +10 mV, separated by 5s depolarizations to pre-defined test potentials.

The normalized voltage-dependent conductance (g_{Ca}), was calculated with the equation: $g_{Ca}=I_{Capeak}/(V-E_{Ca})$, with E_{Ca} equal to the reversal potential for Ca^{2+}, and fitted with a Boltzmann function with variable $V_{1/2}$ (in mV) and k slope (in mV) [16].

The window Ca^{2+} current (I_w) was calculated by multiplying the probability of channel opening ($p_o(V)$ = g_{Ca} x SSI at the respective potential) by the driving force ($V-E_{Ca}$) with E_{Ca} = +62.5 mV.

The intracellular solution for current-clamp measurements was composed of (in mM) 135 K-aspartate, 8 NaCl, 2 $MgCl_2$, 5 EGTA, 20 HEPES, pH 7.4 (with KOH; Sigma-Aldrich). The intracellular solution for voltage-clamp recordings contained (in mM) 135 Cs-$MeSO_3$, 8 NaCl, 2 $MgCl_2$, 5 EGTA and 20 HEPES, pH 7.4 (with CsOH; Sigma-Aldrich). The extracellular solution used for current-clamp measurements was a physiological Tyrode solution containing (in mM): 130 NaCl, 4 KCl, 2 $CaCl_2$, 2 $MgCl_2$, 10 glucose and 10 HEPES; pH 7.4 (with NaOH; Sigma-Aldrich). The extracellular solution used for Ca^{2+} current measurements in voltage-clamp configuration contained (in mM): 135 TEACl, 10 $CaCl_2$, 2 $MgCl_2$, 10 glucose and 10 HEPES, pH 7.4 (with TEA-OH; Sigma Aldrich). L-type currents were recorded in isolation by incubating MCCs in solutions containing 2 μM ω-CTx-MVIIC, 60 nM SNX-482 and 3 μM TTA-P2 to selectively block P/Q-, N-, R- and T-type Ca^{2+} channels which are effectively expressed in MCCs [17] and 300 nM tetrodotoxin (TTX) to selectively block Na^+ currents. In a first series of measurements, L-type current were also determined by subtracting from the total control currents the DHP-insensitive currents remaining after addition of 3 μM nifedipine to the external solution.

Brain slice electrophysiological recordings from SN DA midbrain neurons

Retrograde tracing was performed by stereotaxic surgery in anesthetized mice (isoflurane: induction 4%, maintenance 1.5-3% in 0.35 l/min Oxygen, AbbVie; analgesia by local Lidocaine/Prilocaine (EMLA, AstraZeneca) application on the scull; constant monitoring of body temperature and breathing rate) at the following coordinates (corrected as reported before in [18]): dorsolateral striatum (DLS) bregma: 0.74 mm, lateral: ±2.2 mm, ventral: 2.6 mm); dorsomedial striatum (DMS) bregma: 0.74 mm, lateral: ±1.2 mm, ventral: 2.6 mm). Red beads (Lumaflor, 200 nl) were diluted 1:30 in ACSF and infused with a flow rate of 100 nl/min into the target area using a micro-pump (UMP3-1, World Precision Instruments, 10 µl nanofil syringe, 35-gauge steel needle). 2-4 days after injection patch-clamp experiments were performed. For slice preparation, adult male WT or HET mice were killed by i.p. injections of a ketamine (250 mg/kg, Ketaset, Zoetis) medetomidine-hydrochloride (2.5 mg/kg, Domitor, OrionPharma) mixture and transcardially perfused with ice-cold ACSF containing (in mM): 50 sucrose, 125 NaCl, 2.5 KCl, 25 $NaHCO_3$, 1.25 NaH_2PO_4, 2.5 glucose, 6 $MgCl_2$, 0.1 $CaCl_2$ and 3 kynurenic acid (Sigma-Aldrich), oxygenated with 95%O_2 and 5%CO_2. 250 µm thick coronal midbrain slices between bregma: -2.92 mm and -3.16 mm were obtained using a microtome (VT1200s, Leica). Subsequently slices were left for recovery at 37°C for 1 hour in oxygenated extracellular solution (containing in mM: 22.5 sucrose, 125 NaCl, 3.5 KCl, 25 $NaHCO_3$, 1.25 NaH_2PO_4, 2.5 glucose, 1.2 $MgCl_2$ and 1.2 $CaCl_2$) before being kept at room temperature until recording. For whole-cell current-clamp recordings (at approximately 35°C) slices were transferred into a recording chamber and superfused with oxygenated extracellular solution (flow rate 2-4 ml/min) containing CNQX (20 µM), gabazine (SR95531, 4 µM) and DL-AP5 (10 µM) to block excitatory and inhibitory synaptic transmission. Neurons were visualized using infrared differential interference contrast videomicroscopy with a digital camera (VX55, Till Photonics) connected to an upright microscope (Axioskop 2, FSplus, Zeiss). Retrobeads in retrogradely labelled neurons were visualized by epifluorescence at 546/12nm (X-cite 120PC Q, Excelitas Technologies). Patch pipettes with a resistance of 4-6 MΩ were pulled (DMZ-Universal Puller, Zeitz) from borosilicate glass (GC150TF-10; Harvard Apparatus, Holliston, MA, USA) and filled with internal (containing in mM: 135 K-Gluconate, 5 KCl, 10 HEPES, 0.1 EGTA, 5 $MgCl_2$, 0.075 $CaCl_2$, 5 NaATP, 1 LiGTP, 0.1% neurobiotin, adjusted to a pH of 7.35 with KOH, Osmolarity 290-300 mOsm). Recordings were performed using an EPC-10 patch-clamp amplifier (Heka electronics) with a sampling rate of 20 kHz and a low-pass filter (Bessel, 5 kHz). For analysis, recordings were further digitally filtered at 1 kHz.

Post-hoc immunohistochemical identification of recorded neurons and verification of striatal infusion sites: Midbrain slices containing recorded and neurobiotin-filled neurons as well as forebrain tissue-blocks containing striatal injection sites were immersion-fixed in solution containing 4% paraformaldehyde and 15% picric acid in phosphate buffer solution (PBS) at 4°C overnight. Striatal injection-sites were sliced into serial 100µm coronal sections using a microtome (VT1000S, Leica). Staining was performed on free-floating sections. On the first day, sections were washed in PBS and incubated with blocking solution (10% horse serum, 0.5% Triton X-100% and 0.2% BSA in PBS) at room temperature for 2 h. Afterwards, sections were incubated in carrier solution (1% horse serum, 0.5% Triton X-100% and 0.2% BSA in PBS) containing the primary antibodies (polyclonal rabbit anti-TH, 1:1000, Synaptic Systems Cat# 213 104) overnight at room temperature. On the second day, sections were washed several times in PBS and incubated with the secondary antibody (goat anti-rabbit 488, 1:750, Thermo Fisher Scientific Cat# A-11011 or goat anti-rabbit 405, 1:750, ThermoFisher Scientific Cat# A-31556) and - in case of the midbrain sections - Streptavidin AlexaFluor-488 (1:750, Invitrogen) dissolved in carrier solution overnight at room temperature. On the third day, striatal sections were washed in PBS, stained with 4′,6′-diamidin-2-phenylindol (DAPI, catalog #D1306, Molecular Probes, Invitrogen; 0.02% in PBS) for 5 minutes and washed in PBS for 10 minutes before mounting. Afterwards sections were stored at 4° until confocal microscopy.

Confocal images were taken using a laser-scanning microscope (Nikon Eclipse90i, Nikon GmbH) with the NIS-Elements C program (Nikon software; RRID: SCR_014329) for acquisition. Overview scans of the striatal injection sites were taken using a 4x objective. Images of recorded and therefore neurobiotin-filled neurons were acquired using a 60x immersion objective for post-hoc identification. Overview images of the midbrain were acquired using a 10x or 20x objective.

Brain slice electrophysiological recordings from striatal medium spiny neurons (MSNs)

Male and female Cav1.3^{AG} WT and HET mice (12-17 wk) were used and data was pooled since no significant differences in the parameters were observed. Mice were anaesthetized using isoflurane and sacrificed by cervical dislocation. Brains were rapidly removed and placed immediately into ice cold oxygenated artificial cerebrospinal fluid (aCSF) containing (in mM): 124 NaCl, 3 KCl, 26 $NaHCO_3$, 1 NaH_2PO_4, 2 $CaCl_2$, 1 $MgCl_2$, 16.66 D-(+)-glucose (pH 7.4; 300-310 mOsm/l). 250 µm thick coronal

sections were cut using a vibratome (Leica VT 1200 S) and transferred to a holding chamber containing 37°C warm oxygenated aCSF for 30-60 min. Electrophysiological experiments were performed at room temperature and slices were superfused with oxygenated aCSF throughout the experiment. Internal solution (in mM): 135 $KMeSO_4$, 5 KCl, 0.16 $CaCl_2$, 10 HEPES, 2 Mg-ATP, 0.5 Na-GTP, 5 phosphocreatine-Tris, 5 phosphocreatine-Na, 4 mg/ml biocytin (pH 7.25-7.3; 270-280 mOsml/l). Patch pipettes (3-7 MΩ) were pulled from borosilicate glass capillaries (64-0792, Harvard Apparatus, USA) using a micropipette puller (Sutter Instruments). Recordings were sampled at 20 kHz using an Axon Multiclamp 700 B amplifier (Molecular Devices) and digitized using an Axon Digidata 1440 A (Molecular Devices). Data were analyzed in the Clampfit 10.2 software (Axon Instruments). Dorsal striatal MSNs were identified by the morphology of their soma and their typical electrophysiological characteristics (lack of spontaneous activity, low input resistance, regular spiking [19]). The intrinsic excitability of MSNs was determined in the current clamp configuration by applying 1 s long somatic current injections (-80 to +400 pA; Δ 10 pA).

Microdialysis in freely moving mice

Under isoflurane anesthesia (at 5 % and 1.5 % for induction and maintenance, respectively), a commercially available guide cannula (MAB 4.15.IC, Microbiotech, Sweden) was implanted above the right dorsomedial striatum (bregma: 0.74 mm, lateral: 1.2 mm, ventral: 1.9 mm) and closed with a dummy probe as described previously [20]. After surgery, animals received buprenorphine (0.5 mg/kg s.c.) and meloxicam (1.0 mg/kg p.o. via the drinking water) for analgesic care. They were allowed to recover for 5-7 days while being single-housed and habituated to the experimental procedures.

About 36-48 hours before the experiment started, animals were kept in special in-house designed microdialysis cages (22 × 30 × 31 cm) placed in the experimental room. Mice had free access to food and water during the whole experiment. On the day before the experiment the dummy cannula was replaced by a microdialysis probe (MAB 4.15.1 PES, Microbiotech, Sweden) that extended the guide cannula by 1 mm, thus, reaching into the dorsomedial striatum. Microdialysis probes were connected to a microinfusion pump (CMA, Stockholm, Sweden) and a swivel-tether system via polyethylene tubing. Probes were constantly perfused with artificial cerebrospinal fluid (aCSF; consisting of in mM: 140 NaCl, 3.0 KCl, 1.2 $CaCl_2$, 1.0 $MgCl_2$, 1.0 Na_2HPO_4, pH 7.4) at a flow rate of 0.5 µl/min overnight and 1.0 µl/min

during sample collection, respectively. After an equilibration period of 60 min, several consecutive microdialysis fractions were collected every 10 min in pre-cooled microtubes containing antioxidative protection solution (in mM: 0.27 Na_2EDTA, 100 acetic acid, 0.0125 ascorbic acid), vortexed and immediately frozen at -80 °C until further analysis. The first six microdialysis fractions were sampled in the home cage as baseline values. Thereafter, animals were transferred to a novel cage (22 x 30 x 31 cm plexiglass cage without bedding material, swiped with 70% ethanol). After a 3 h exposure, animals were returned back to their home cages and another twelve 10 min microdialysates were collected. Finally, microdialysis probes were perfused with high (100 mM) KCl containing aCSF in order to elicit local depolarization for testing release capacity and functionality of the microdialysis systems. Finally, animals were euthanised with an overdose of thiopental, decapitated and their brains were removed. The localization of the microdialysis probe was verified by observing cresyl violet stained coronal sections (40 µm) under a light microscope. Only subjects where the microdialysis probe has been correctly localized in the respective target brain area were included in the analysis.

Quantification of dopamine in microdialysates

Quantification of dopamine in microdialysates was performed using a high-performance liquid chromatography (HPLC) system with electrochemical detection as previously described [20]. The HPLC system consisted of a Shimadzu (Kyoto, Japan) system equipped with a controller (CBM-20A), degassing unit (DGU-20A3R) and micro HPLC pump (LC-20ADXR) operated at a flow rate of 0.055 mL/min. The system was combined with an electrochemical detector (Decade II, Antec Scientific, Netherlands) that was coupled directly to a 2 mm glassy carbon working electrode (SenCell, Antec Scientific, Netherlands). Chromatographic separation of analytes was performed on an ACQUITY UPLC BEH C18 column (100 x 1 mm, 1.7 µm spherical particles, Waters Milford, Massachusetts, United States). Detection was carried out at 35°C with an applied potential of +480 mV vs. Ag/AgCl reference electrode. Samples of 5 µl were automatically injected by a SIL-20ACXR refrigerated autosampler (Shimadzu, Japan). The mobile phase consisted of 7% (v/v) acetonitrile, 150 mM sodium acetate, 20 mM citric acid, 10 mM sodium decane-sulfonate, 0.5 mM Na_2EDTA at a pH of 5.5 that was filtered (0.2 µm) before use. Evaluation of dopamine was carried out by comparing peak areas of samples with a set of external standard solutions (range between 100 pM - 5 nM) with Lab Solution chromatography

software (LabSolution CS, Shimadzu, Japan). The detection limit for dopamine was 0.25 fmol/5 µl sample injection, respectively, at a signal to noise ratio of 3.

Behavioral analysis

Animals were single-housed for at least two weeks prior the start of behavioral testing, which was performed in parallel with male WT and mutant animals of the same litter (age ~12-14 weeks at the start of testing). The experimenter was blinded to the genotype. To minimize the used number of animals, a battery of tests was conducted with the same animals starting with the least invasive one. Behaviors were analyzed from pre-recorded videos either manually or automatically using ANY-maze behavioral tracking software (v 7.10; Stoelting, Dublin, Ireland).

Home cage activity

The spontaneous locomotor activity of all 3 genotypes was assessed in their home cage as previously described [21]. Measurement was started at the beginning of the light cycle (07:00) after an initial habituation to the new home cage (12-16 hours). Locomotion was recorded in 1 min intervals for 48 h including two light and two dark cycles by an automated system (Inframot, TSE, Bad Homburg, Germany). The system monitored the activity of the mice by sensing the body heat image, i.e., infra-red radiation, and its spatial displacement over time. Data of 1 min bins were pooled to 1 h intervals.

Open field

The open field consisted of a plastic box (41 x 41 x 41 cm). Illumination at floor level was 150 lux. No bedding was used. Mice were individually placed into the periphery of the open field with their nose facing the wall. The behavior was tracked for 10 min as described previously [22]. The time spent in the central or peripheral zone, the number of entries into the central or peripheral zone, overall distance travelled by the mice, time spent mobile/immobile and the average velocity during the mobile phase were quantified using ANY-maze software.

Elevated pluz maze

Mice were placed into a plus maze elevated 72cm from the ground, consisting of two closed arms (30 x 5cm) illuminated with <10 lux white light, and two open arms (30 x 5 cm) illuminated with white light at 100 lux, as previously described [22]. The behavior was tracked for 5 min. The test started by placing the mouse on the center platform (5 x 5 cm) facing a closed arm. The behavioral parameters measured using the ANY-maze software included the time spent in the open/closed arms, entries to the open/closed arms, total distance travelled, mobile/immobility time as well as average velocity during the mobile phase.

Light dark box test

The light/dark test was performed in the open field box, but divided into a light arena illuminated at 400 lux, and a closed dark chamber (black box) illuminated at <10 lux. The dark box was accessible through a small door (assigned as transition zone). Mice were placed at the door of the dark chamber with the nose facing the dark compartment and when it entered the dark chamber, it was allowed to explore the full arena for 10min. Behavior was quantified manually. Parameters measured included time spent in as well as number of entries to the light arena as described previously [22].

Marble burying test

The open field test arena (described above) was divided into half using a cardboard. On one half of the arena bedding (similar to the home cage) 20 glass marbles (diameter: 15 mm) were added equidistant in a 4 × 5 arrangement on top of a 18 cm layer of bedding material, with slight modification from the protocol developed by Thomas at al. (2009)[23]. Mice were tested for 30 min. Marbles were thoroughly cleaned with acetic acid solution (0.1%) between individual subject mice. Testing was performed on three consecutive days and the social context was manipulated by the presence or absence of social odors. On the first day, fresh bedding was used (data shown in Figure 2C). On the second day, soiled bedding from an unfamiliar cage housing mice of the same sex as the subject mouse was added on top of the layer of fresh bedding. Finally, on the third day, fresh bedding was used again. On each of the 3

days, marbles that were covered at least 4/5th or more with the bedding were classified as "completely buried" and were taken into analysis. Data from days 2 and 3 were comparable to day 1 (not shown).

Grooming/Rearing

Mice were placed in the open field as described in the "open field test" section. The floor consisted of fresh bedding similar to the home cage. The mice were allowed to freely explore the arena for 10min.The testing was conducted for 3 days wherein the first day was classified as "novel environment" and the third day as "familiar environment". Grooming and rearing bouts and time were scored manually.

3-chamber sociability test

The testing apparatus (dimension 60 x 40 x 22 cm) consisted of a 3-chambered arena (2 lateral chambers and 1 middle chamber) with each chamber measuring 20 x 40 x 22 cm (Cat # 46553, Ugo Basile, Itlay). Each of the 2 partition walls consisted of a small door for the mouse to enter/exit. On the first day (habituation), the experimental mice (to be tested) were placed in the central compartment right against the middle part of the proximal wall (not having doors) with its head facing the central zone. The doors were open and the mouse was allowed to explore the entire arena for 10min.

On the second day (testing) the experimental mice were placed in the central compartment (as described on the habituation day). The time that the mice spent in each of the lateral compartments was calculated on a real time basis. At the end of 10min, the experimental mouse was gently (without picking up) brought to the central compartment and both doors were closed. 2 metal grid enclosures (each 15cm tall with an internal diameter of 7cm, Cat # 46503-003) were now placed in each of the lateral chambers. A stimulus mouse (of the same sex and genotype, but lower in body weight) was placed in one of the grid enclosure while the other grid chamber was kept empty. The grid enclosure with the stimulus mouse was placed in the lateral chamber where the experimental mouse spent less time during the first 10min of the testing day (social chamber) while the other empty grid enclosure was placed in the other lateral compartment (non-social chamber). Both the doors were removed and the experimental mouse was now allowed to explore the arena for an additional 10min. The time spent in each of the compartment as well as nose-to-grid interaction time in each of the chamber was quantified manually.

In vivo *isradipine rescue experiments*

Male and female WT and HET mice were single-housed for at least two weeks prior the EPM test (age: 10-22 wk). Experiments were performed in parallel with both genotypes. Male mice received 0.5-1 ml strawberry fruit yoghurt twice a day (at ~9:00 and 17:00) for one week. At the end of this "training week", all mice reproducibly ate up the 0.5-1 ml per feeding session. In the following week, on day 0 mice still received pure yoghurt and were tested in the open field to confirm the presence of the HET hyperlocomotive phenotype in the test cohorts. Oral isradipine administration started on day 1 and mice were offered 0.5-1 mg isradipine (vascal® uno 5 mg, extended-release formulation, registration number: 29421.01.00) mixed into 0.5-1 ml yoghurt twice a day or yoghurt only (vehicle group) for two consecutive days (~9:00 and ~17:00). On day 3, mice received the last dose in the morning and were tested in the EPM 4-5 hours later to determine potential drug-induced effects on locomotion. To test effects of lower doses and to confirm hyperlocomotion in females as well, female mice were first tested in the open field. Then, mice received 0.2 ml yoghurt 3-times a day at ~8:00, 16:00 and 24:00 ("training week"). Upon successful completion of the training, mice received either yoghurt (vehicle group) or 0.1 mg ISR mixed into yoghurt three times a day as in the training week. On day 3, after the morning dose, mice were tested in the EPM. Plasma was taken immediately after the EPM and isradipine plasma concentrations were determined with a liquid chromatography-tandem mass spectrometry (LC-MS/MS) method.

Plasma isradipine measurements

Isradipine concentrations were determined with a liquid chromatography-tandem mass spectrometry (LC-MS/MS) method. An isotopically labeled analogue was used as internal standard (isradipine-D3). Water, methanol, acetic acid and acetonitrile (all HPLC grade) were purchased from Honywell (Seelze, Germany). Isradipine and isradipine-D3 were obtained from Toronto Research Chemicals (Toronto, Canada). The LC-MS/MS system consisted of a Waters ACQUITY UPLC H-Class PLUS Bio (Waters, Manchester, UK) and a QTrap 6500+ mass spectrometer (Sciex, Framingham, MA, USA). Chromatographic separations were accomplished on a reversed-phase column (Kinetex 2.6 µm Biphenyl 100 Å, 100 × 2.1 mm, Phenomenex, Aschaffenburg, Germany) using a 1-minute linear gradient of 10-98% methanol in aqueous 0.5% acetic acid solution (v/v). The gradient program was started 1 min

after the injection. The flow rate was set to 200 µl/min. The column temperature was held at 50°C. The injection volume was 10 µl. Mass spectrometry detection was performed with electrospray ionization in negative ion mode. Multiple reaction monitoring was carried out using the precursor-to-product ion transitions *m/z* 370.1 to 250.1 and *m/z* 370.1 to 119.1 for isradipine and *m/z* 373.1 to 253.1 and *m/z* 373.1 to 119.1 for isradipine-D3. Sample preparation involved a protein precipitation step. Before use, plasma samples were allowed to equilibrate to room temperature. A 50 µl-aliquot of the sample was mixed with 5 µl of internal standard solution (30 ng/ml) and 95 µl of methanol. The mixture was vortexed and centrifuged at 4,600×g for 5min. The obtained supernatant was submitted to LC-MS/MS analysis. Calibration was accomplished by donating isradipine-free mouse plasma with known concentrations of isradipne ranging from 0.020 to 100 ng/ml. The limit of quantification was 0.050 ng/ml.

References

1. Platzer, J., et al., *Congenital deafness and sinoatrial node dysfunction in mice lacking class D L-type Ca^{2+} channels.* Cell, 2000. **102**(1): p. 89-97.
2. Schneider, C.A., W.S. Rasband, and K.W. Eliceiri, *NIH Image to ImageJ: 25 years of image analysis.* Nat Methods, 2012. **9**(7): p. 671-5.
3. Mastrolia, V., et al., *Loss of alpha2delta-1 Calcium Channel Subunit Function Increases the Susceptibility for Diabetes.* Diabetes, 2017. **66**(4): p. 897-907.
4. Paxinos, G. and K.B.J. Franklin, *The Mouse Brain in Stereotaxic Coordinates*. Fourth edition. ed. Vol. Second edition. 2001, San Diego, Calif.; London: Academic Press. 296.
5. Fu, Y., et al., *A cytoarchitectonic and chemoarchitectonic analysis of the dopamine cell groups in the substantia nigra, ventral tegmental area, and retrorubral field in the mouse.* Brain Struct Funct, 2012. **217**(2): p. 591-612.
6. Franklin, K.B.J. and G. Paxinos, *The Mouse Brain in Stereotaxic Coordinates, 3rd Edition.* Academic Press, 2007.
7. Pinggera, A., et al., *CACNA1D De Novo Mutations in Autism Spectrum Disorders Activate Cav1.3 L-Type Calcium Channels.* Biol Psychiatry, 2015. **77**(9): p. 816-22.
8. Ortner, N.J., et al., *Lower affinity of isradipine for L-type Ca^{2+} channels during substantia nigra dopamine neuron-like activity: implications for neuroprotection in Parkinson's disease.* J Neurosci, 2017.
9. Lieb, A., N. Ortner, and J. Striessnig, *C-terminal modulatory domain controls coupling of voltage-sensing to pore opening in Cav1.3 L-type Ca(2+) channels.* Biophys J, 2014. **106**(7): p. 1467-75.
10. Hofer, N.T., et al., *Biophysical classification of a CACNA1D de novo mutation as a high-risk mutation for a severe neurodevelopmental disorder.* Mol Autism, 2020. **11**: p. 4.
11. Marcantoni, A., et al., *PDE type-4 inhibition increases L-type Ca(2+) currents, action potential firing, and quantal size of exocytosis in mouse chromaffin cells.* Pflugers Arch, 2009. **457**(5): p. 1093-110.
12. Vandael, D.H., et al., *Ca(V)1.3-driven SK channel activation regulates pacemaking and spike frequency adaptation in mouse chromaffin cells.* J Neurosci, 2012. **32**(46): p. 16345-59.
13. Marcantoni, A., et al., *Loss of Cav1.3 channels reveals the critical role of L-type and BK channel coupling in pacemaking mouse adrenal chromaffin cells.* J Neurosci, 2010. **30**(2): p. 491-504.
14. Calorio, C., et al., *Impaired chromaffin cells excitability and exocytosis in autistic Timothy syndrome TS2-neo mouse rescued by L-type calcium channel blockers.* J Physiol, 2019.
15. Cesetti, T., et al., *Opposite action of beta1- and beta2-adrenergic receptors on Ca(V)1 L-channel current in rat adrenal chromaffin cells.* J Neurosci, 2003. **23**(1): p. 73-83.
16. Guarina, L., et al., *Low pHo boosts burst firing and catecholamine release by blocking TASK-1 and BK channels while preserving Cav1 channels in mouse chromaffin cells.* J Physiol, 2017. **595**(8): p. 2587-2609.
17. Mahapatra, S., et al., *Calcium channel types contributing to chromaffin cell excitability, exocytosis and endocytosis.* Cell Calcium, 2012. **51**(3-4): p. 321-30.
18. Lammel, S., et al., *Unique properties of mesoprefrontal neurons within a dual mesocorticolimbic dopamine system.* Neuron, 2008. **57**(5): p. 760-73.
19. Ibanez-Sandoval, O., et al., *Electrophysiological and morphological characteristics and synaptic connectivity of tyrosine hydroxylase-expressing neurons in adult mouse striatum.* J Neurosci, 2010. **30**(20): p. 6999-7016.
20. Ebner, K., et al., *The Novel Analogue of Modafinil CE-158 Protects Social Memory against Interference and Triggers the Release of Dopamine in the Nucleus Accumbens of Mice.* Biomolecules, 2022. **12**(4).

21. Singewald, N., et al., *Magnesium-deficient diet alters depression- and anxiety-related behavior in mice--influence of desipramine and Hypericum perforatum extract.* Neuropharmacology, 2004. **47**(8): p. 1189-97.
22. Rooney, S., et al., *Neuroinflammatory alterations in trait anxiety: modulatory effects of minocycline.* Transl Psychiatry, 2020. **10**(1): p. 256.
23. Thomas, A., et al., *Marble burying reflects a repetitive and perseverative behavior more than novelty-induced anxiety.* Psychopharmacology (Berl), 2009. **204**(2): p. 361-73.

Supplemental Figures and Tables

	hCav1.3L WT	n/N	hCav1.3L A749G	n/N	p WT vs AG	hCav1.3L G407R	n/N	p WT vs GR
$V_{0.5}$ [mV]	-0.6 ± 0.9	30/5	-14.1 ± 0.6	28/3	<0.001 ***	-6.6 ± 0.6	20/4	<0.001 ***
k [mV]	9.0 ± 0.1	30/5	6.4 ± 0.2	28/3	<0.001 ***	8.3 ± 0.2	20/4	0.0058 **
V_{max} [mV]	13.8 ± 0.8	30/5	-1.0 ± 0.8	28/3	<0.001 ***	6.8 ± 0.7	20/4	<0.001 ***
Act thresh [mV]	-34.6 ± 0.6	30/5	-37.1 ± 0.4	28/3	0.0027 **	-37.9 ± 0.8	20/4	<0.001 ***

Supplemental Table 1: Voltage-dependence of activation of hCav1.3$_L$ WT, A749G and G407R channel complexes (β3, α2δ1) in tsA-201 cells. Transiently transfected cells were depolarized for 30 ms from a holding potential of -89 mV to different test potentials (Δ 5 mV) with 15 mM Ca^{2+} as charge carrier. The voltage-dependence of activation of both mutants was shifted towards more negative potentials as reported in the original study describing these variants [1]. Date are given as mean ± SEM for the indicated number of cells (n) and independent transfections (N). Statistical significance was determined by a one-way ANOVA ($V_{0.5}$, k, V_{max}, act thresh: $p<0.001$) with Dunnett's multiple comparisons post-hoc test as indicated in the Table. $V_{0.5}$, voltage of half-maximal activation; k, slope factor; V_{max}, voltage of maximal activation; act thresh, activation threshold (voltage at which 5 % of peak I_{Ca} was conducted)

	DLS projecting lateral SN DA neurons					DMS projecting medial SN DA neurons				
**Parameter**	**WT**	**n/N**	**HET**	**n/N**	**p**	**WT**	**n/N**	**HET**	**n/N**	**p**
On-cell frequency [Hz]	2.61 ± 0.39	11/5	2.71 ± 0.16	19/4	0.2694	1.84 ± 0.16	20/4	2.50 ± 0.19	28/4	**0.0069 ****
Whole-cell frequency [Hz]	2.68 ± 0.35	11/5	2.77 ± 0.20	19/4	0.8217	1.71 ± 0.12	22/4	2.86 ± 0.29	28/4	**<0.001 *****
Spike threshold [mV]	-31.09 ± 1.83	11/5	-30.72 ± 1.10	19/4	0.6719	-28.04 ± 1.22	22/4	-27.58 ± 0.93	28/4	0.9460
Spike width [ms]	3.31 ± 0.17	11/5	3.26 ± 0.11	19/4	0.7948	3.89 ± 0.14	22/4	3.88 ± 0.16	28/4	0.4790
miniAHP [mV]	-56.81 ± 1.68	11/5	-56.49 ± 3.54	19/4	0.8990	-55.39 ± 0.91	22/4	-54.24 ± 1.02	28/4	0.9306
CV [%]	6.53 ± 0.77	11/5	7.13 ± 0.82	19/4	0.8990	10.04 ± 0.96	22/4	10.05 ± 1.46	28/4	0.2317
Capacitance [pF]	109.3 ± 9.36	11/5	80.26 ± 4.62	19/4	**0.0124 ***	114.8 ± 7.41	22/4	109.5 ± 6.18	28/4	0.8883
Rm [MΩ]	388.8 ± 71.17	11/5	445.1 ± 37.92	19/4	0.4495	633.5 ± 46.21	22/4	525.3 ± 40.22	28/4	0.0830
Sag component [mV]	14.91 ± 1.28	11/5	15.31 ± 1.13	19/4	0.8213	13.31 ± 0.87	22/4	16.02 ± 0.91	28/4	**0.0395 ***
Rebound delay [ms]	346.3 ± 65.68	11/5	224.4 ±41.87	19/4	0.1853	676.4 ± 85.54	22/4	394.7 ± 43.90	28/4	**0.0027 ****

Supplemental Table 2: Parameters obtained by whole-cell patch-clamp recordings from SN DA neurons in brain slices from male WT and HET Cav1.3^{AG} mice. Retrograde labeling through redbead infusion into the dorsolateral (DLS) or dorsomedial striatum (DMS) enabled projecting-specific recoding of DLS-projecting lateral or DMS-projecting medial SN DA neurons. The autonomous pacemaker frequency was obtained in the on-cell configuration and after whole-cell break-in (no significant difference between both configurations). Data are given as mean ± SEM for the indicated number of cells (n) and preparations (N). Statistical significance was determined using unpaired Student's t-test or Mann-Whitney test: *** p<0.001, ** p<0.01, * p<0.5.

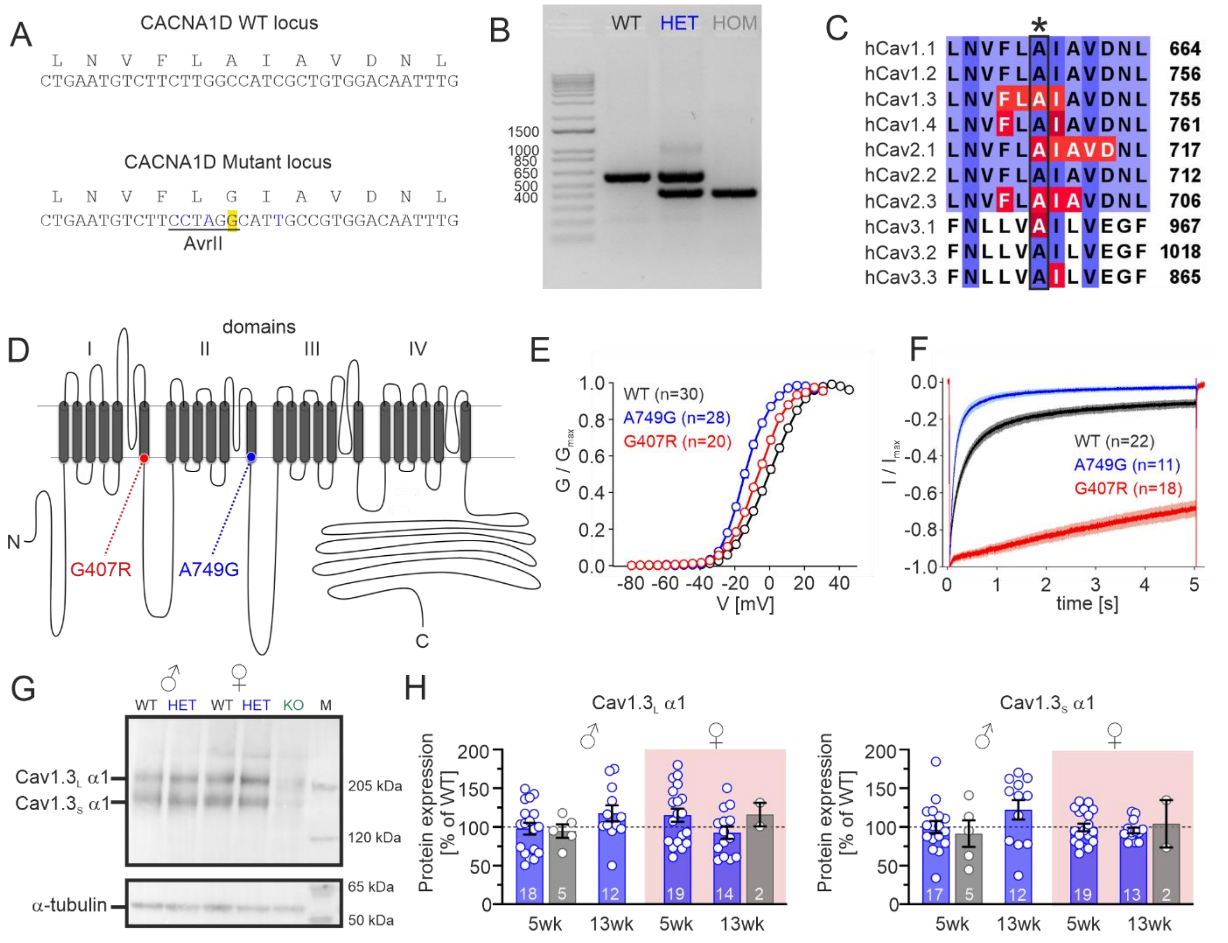

Supplemental Figure 1: Genomic *CACNA1D* locus of WT and Cav1.3^{AG} mutant mice, variant-specific gating changes and similar brain Cav1.3 α1 protein levels. A, *CACNA1D* gene locus before (WT, top) and after CRISPR/Cas9 targeting (Mutant, bottom). The WT alanine at position 749 (reference sequence EU_363339) was mutated to an glycine. Mutated nucleotides are indicated in bolt blue. An addition AvrII restriction site was introduced for sequencing purpose (silent mutation). **B,** Agarose gel picture showing the band pattern of the PCR genotyping approach (for details see Methods). WT: 974 bp + 600 bp; HET: 974 bp, 600 bp + 412 bp; HOM: 974 bp + 412 bp; The 974 bp band is barely visible. Marker: 1 Kb Plus DNA Ladder (Thermo Fisher; Cat#10787026). **C,** Sequence alignment of the targeted domain II S6 segment of all human voltage-gated Ca^{2+} channel α1 subunits with pathogenic mutations indicated in red (modified from [2]). The affected alanine is framed in a box and marked with an asterisk. Conservation of amino acids among channels is indicated in blue, residues with described pathogenic mutations are shown in red. For Uniprot IDs please see [2]. **D,** Topology of the pore-forming Cav1.3 α1-subunit, consisting of four domains which are built by six transmembrane segments (TM). The position of the G407R (red) and A749G (blue) point mutations within TM6 of domain I or II, respectively, are indicated. **E,F,** Whole-cell patch-clamp experiments showing the gating properties of human Cav1.3$_L$

WT, A749G and G407R channels in tsA-201 cells (with β3 and α2δ1; 15 mM Ca^{2+}). Comparable gating changes to the original study were observed for both, A749G and G407R mutant channels [1]. **E,** Conductance-voltage (G-V) curves show the mean ± SEM for the indicated number (n) of cells. For parameters and statistics see Supplemental Table 1. **F,** Averaged traces (mean ± SEM) of the I_{Ca} inactivation kinetics of WT and mutant channels during a 5-s long depolarization to V_{max}. **G,** Representative Western Blot of membrane preparations from male and female $Cav1.3^{AG}$ WT and HET mice (5 wk) and a Cav1.3 knockout control (KO; 40 wk). The used anti-Cav1.3 antibody recognizes both, C-terminally long ($Cav1.3_L$) and short α1 splice variants ($Cav1.3_S$, [3, 4]) as indicated. M = Marker. For Cav1.3 α1 and tubulin different exposure times are shown. **H,** Quantification of $Cav1.3_L$ and $Cav1.3_S$ α1 protein expression in brain membrane preparations (N=2-4 animals/group) from male and female HET (blue) or HOM (grey) $Cav1.3^{AG}$ mice at the indicated age showed no significant differences. For details see Supplemental Methods. Signals were normalized to α-tubulin and given as the mean ± SEM in % of WT.

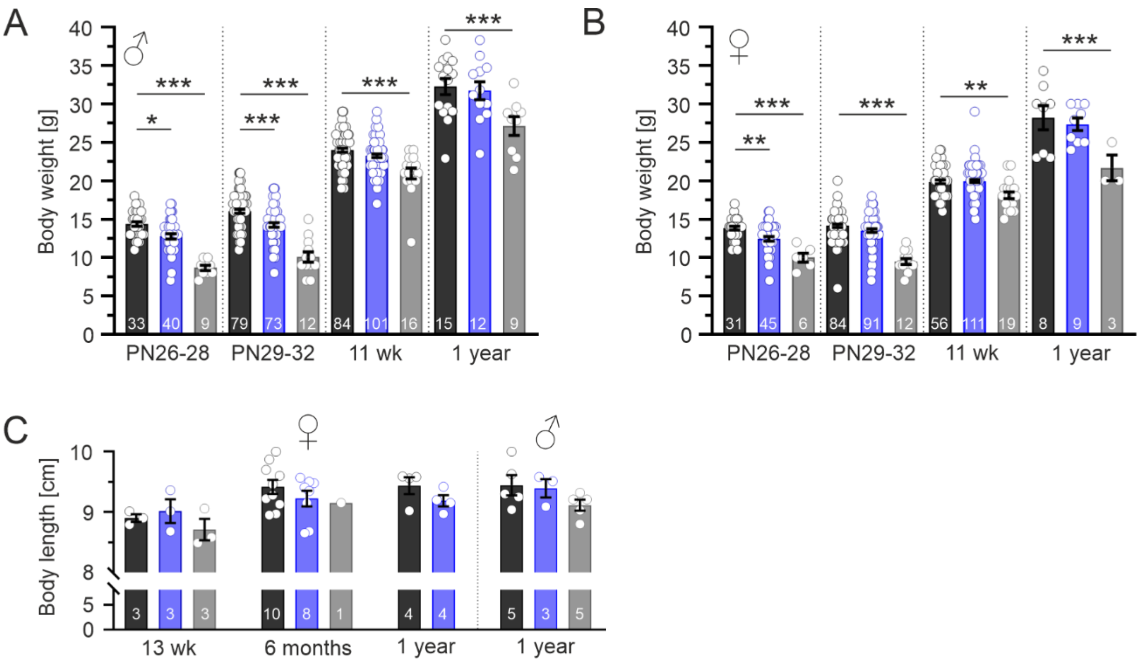

Supplemental Figure 2: Cav1.3^{AG} mutant mice show a delayed (HET) or reduced (HOM) gain of body weight. A,B, Body weight of male (**A**) and female (**B**) WT and mutant Cav1.3^{AG} mice for the indicated age and number of animals. PN = postnatal day. Data are given as mean ± SEM. Statistical significance was determined using two-way ANOVA (male Age: $F_{3,471}$=585.0, p<0.001; Genotype: $F_{2,471}$=63.49, p<0.001; female Interaction: $F_{6,463}$=3.618, p=0.0016; Age: $F_{3,463}$=343.2, p<0.001; Genotype: $F_{2,463}$=39.95, p<0.001) with Dunnett's multiple comparisons post-hoc test as indicated in the graph: *** p<0.001, ** p<0.01, * p<0.5. **C,** Body length measured from nose tip to tail base of male and female WT and mutant Cav1.3^{AG} mice at the indicated age showed no statistically significant differences.

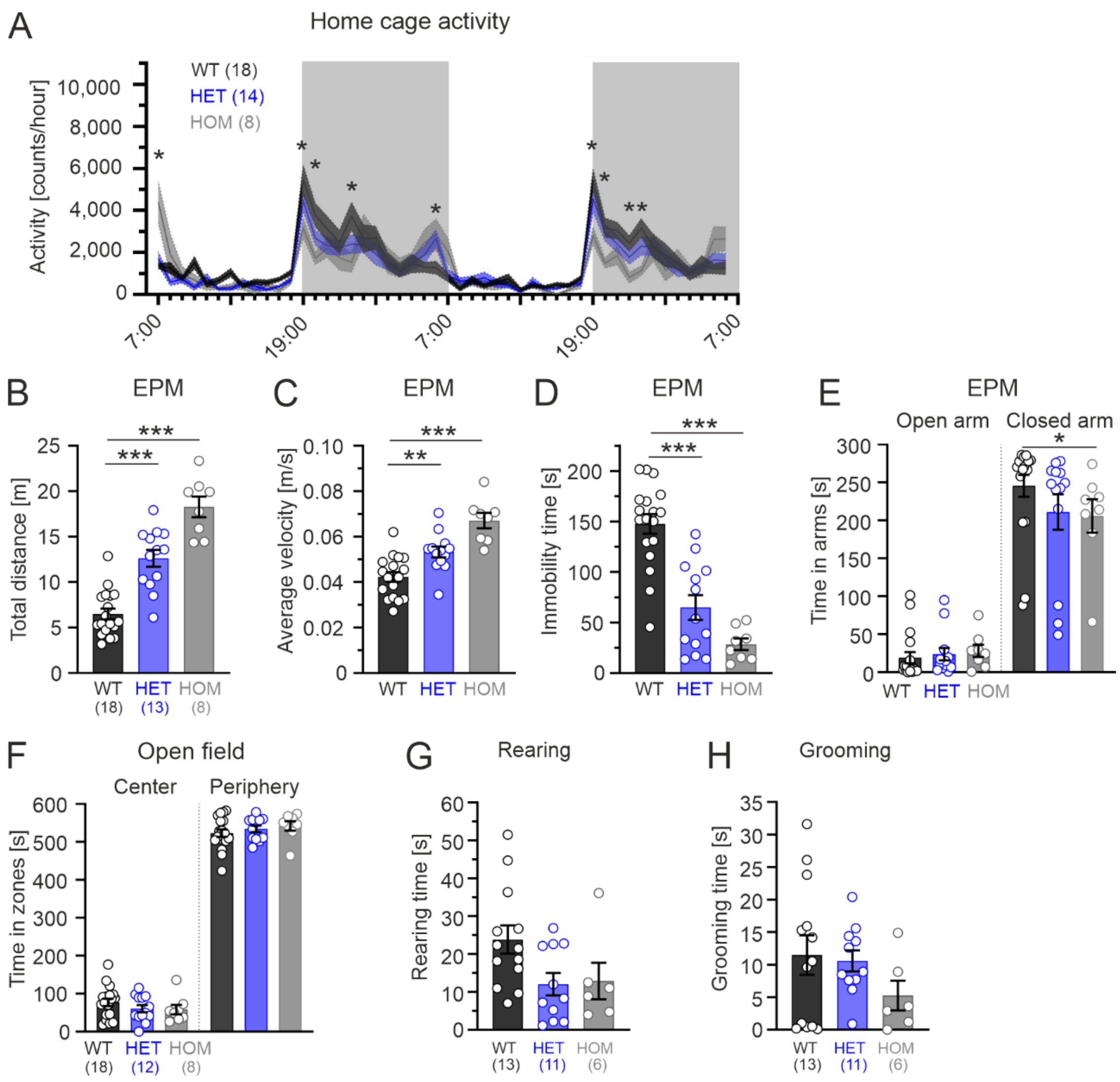

Supplemental Figure 3: Cav1.3^{AG} mutant mice were hyperlocomotive in the elevated plus maze but not in their home cage. Behavioral tests were performed with adult male WT (black), HET (blue) or HOM (grey) mice. Data are given as mean ± SEM for the indicated number of animals. Statistical significance was determined using one-way ANOVA (**B,C,D,F,G**) or Kruskal-Wallis (**E,G**) with Dunnett's or Dunn's multiple comparison post-hoc test, respectively, as indicated in the graphs. *** $p<0.001$, ** $p<0.01$, * $p<0.05$. **A,** Mice were habituated to the new home cage for 12-16 h and subsequently horizontal and vertical activity counts were recorded in the home cage during two consecutive light and dark phases (grey rectangles represent dark phases). A repeated measures ANOVA revealed a significant time X genotype effect; $F(94, 1739) = 2.3256$, $p<0.001$. Fischer-LSD post hoc test revealed that baseline activity was reduced during the first half of the dark (active) phase in HOM mutants, while it did not differ among genotypes during the light (inactive) phase. **B-D,** In line with the data from the open field (Figure 2A), Cav1.3^{AG} mutant mice displayed a significantly increased distance travelled (**B**,

p<0.001) and average velocity during mobile time (**C**, p<0.001), as well as a significant decrease in time spent immobile (**D**, p<0.001). **E**, No significant difference in the time spent in the open (“anxiogenic”) arm was observed, while a slight reduction of closed (“safe”) arm time was found for HOM mutant mice (p=0.0223). **F**, Likewise, time spent in the center (“anxiogenic”) and periphery (“safe”) zone of the open field showed no significant differences. **G,H,** Quantification of the time spent rearing and grooming in a novel environment during a 10 min time period did not show significant differences among genotypes. When repeated in a familiar environment, again no differences were observed (data not shown).

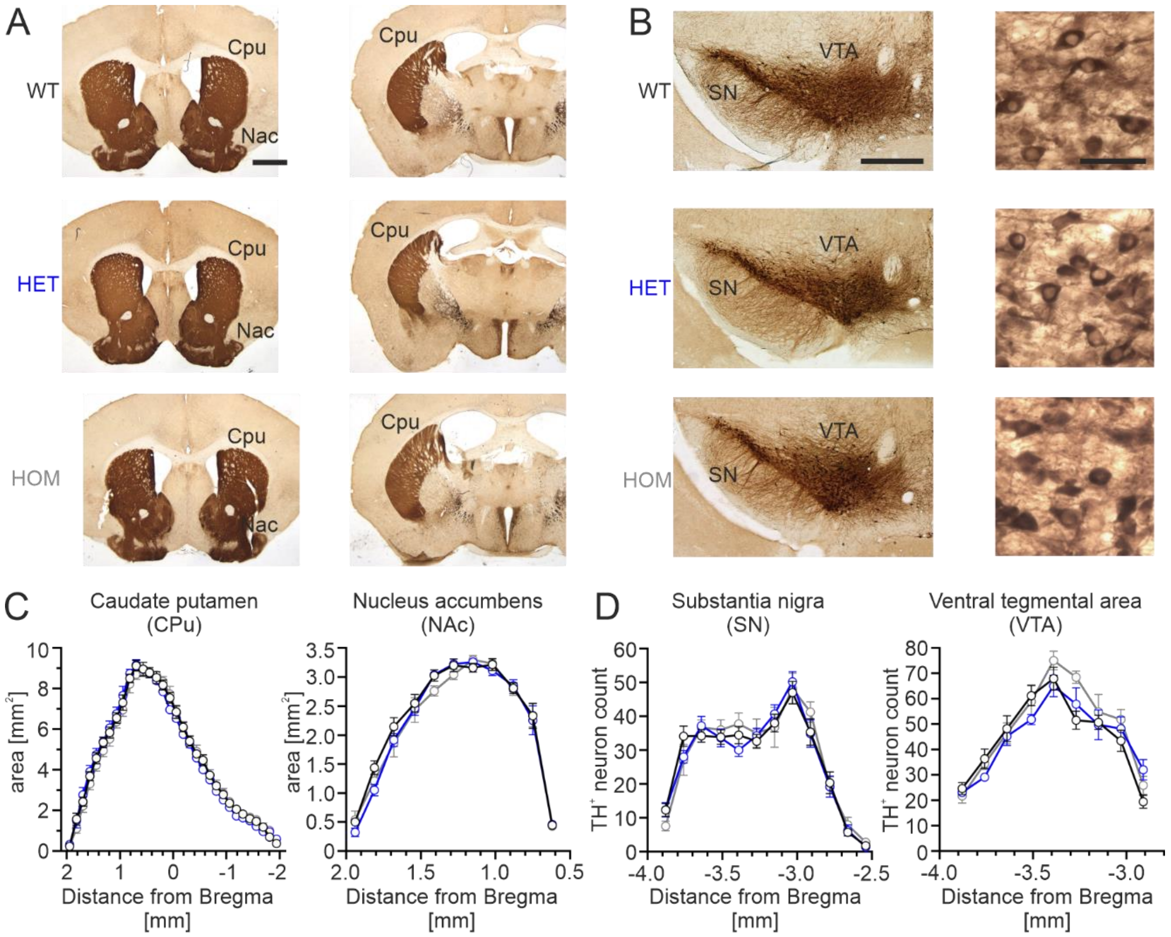

Supplemental Figure 4: No difference in striatal volume and DA midbrain neuron number in Cav1.3^{AG} mutant mice. Data represent mean ± SEM. To visualize the striatum and DA midbrain neurons, 40 µm thick coronal sections between Bregma 1.94 and -3.88 mm (according to The Mouse Brain in Stereotaxic Coordinates Third Edition [5]) from adult male Cav1.3^{AG} WT and mutant mice (~12 wk) were stained against tyrosine-hydroxylase (TH; high density of axonal terminals from DA midbrain neurons within the striatum). **A,** Representative pictures of the striatum from WT (top), HET (middle) and HOM (bottom) Cav1.3^{AG} mice at Bregma 1.18 mm (left) or -0.70 mm (right). Scale bar: 1 mm. **B,** Representative pictures showing the substantia nigra (SN) and ventral tegmental area (VTA) at Bregma -3.08 mm from WT (top), HET (middle) and HOM (bottom) Cav1.3^{AG} mice. Left: Overview (scale bar: 500 µm). Right: Enlargement showing individual TH$^+$ cells in the medial SN (scale bar: 50 µm). **C,** Plotting the quantified area of each section versus the coordinates along the rostro-caudal axis revealed no differences in mutant mice compared to WT (WT n=10; HET n=6; HOM n=5). **D,** Plotting the TH$^+$ neuron count determined in both hemispheres of each section versus the coordinates along the rostro-caudal axis revealed a similar distribution (WT n=10; HET n=7; HOM n=5).

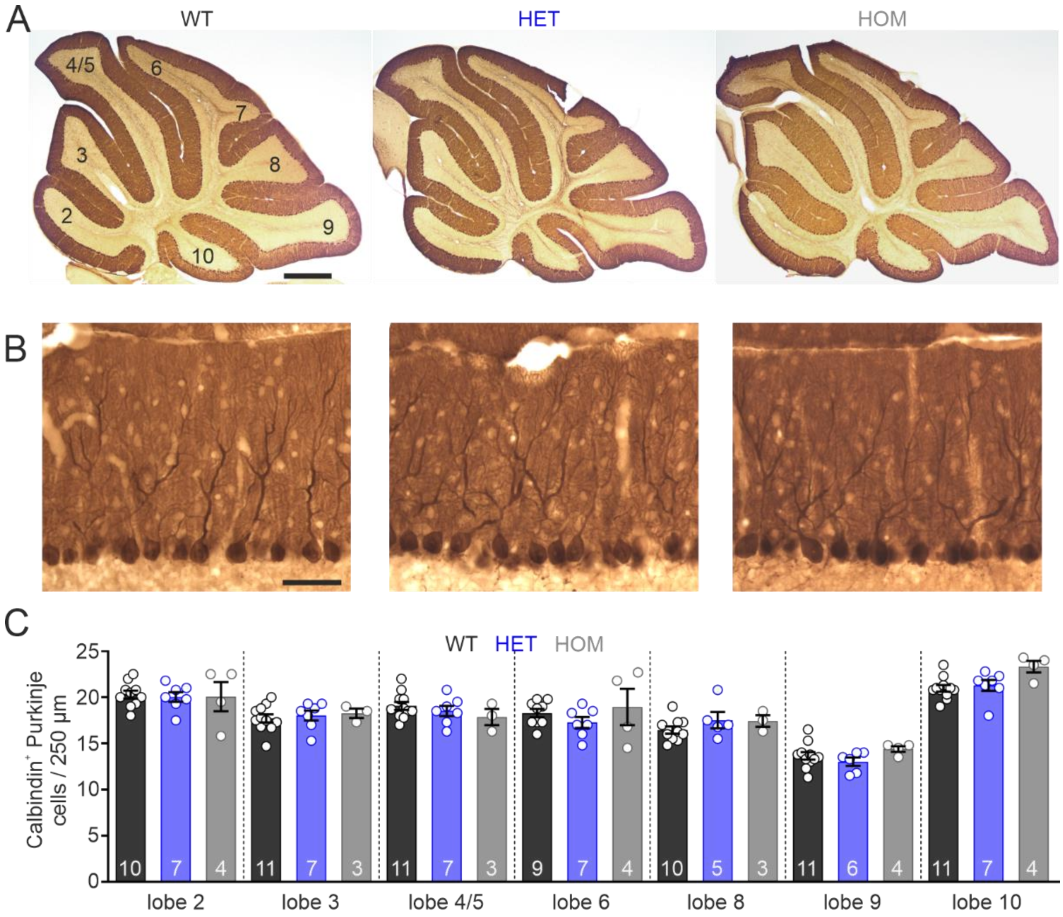

Supplemental Figure 5: Cerebellar calbindin⁺ Purkinje cell density was unchanged in Cav1.3^{AG} mutant mice. 40 µm thick sagittal sections around the midline from adult male Cav1.3^{AG} WT and mutant mice (~12 wk) were stained against calbindin D-28k to visualize cerebellar Purkinje cells. **A-B,** Representative pictures showing an overview (**A**, scale bar: 500 µm) and individual calbindin^{+} Purkinje cells (**B**, scale bar: 50 µm). **C,** The number of calbindin^{+} cerebellar Purkinje cells in the indicated lobes was determined on a 250 µm long stretch (same site per lobe analyzed in all animals). Datapoints represent the mean cell number from 3-4 sagittal sections around the midline per animal (mean ± SEM). Two-way ANOVA analysis did not show a statistical significance for the genotype or lobe-genotype interaction (lobe: $F_{6,124}$=48.25, p<0.0001).

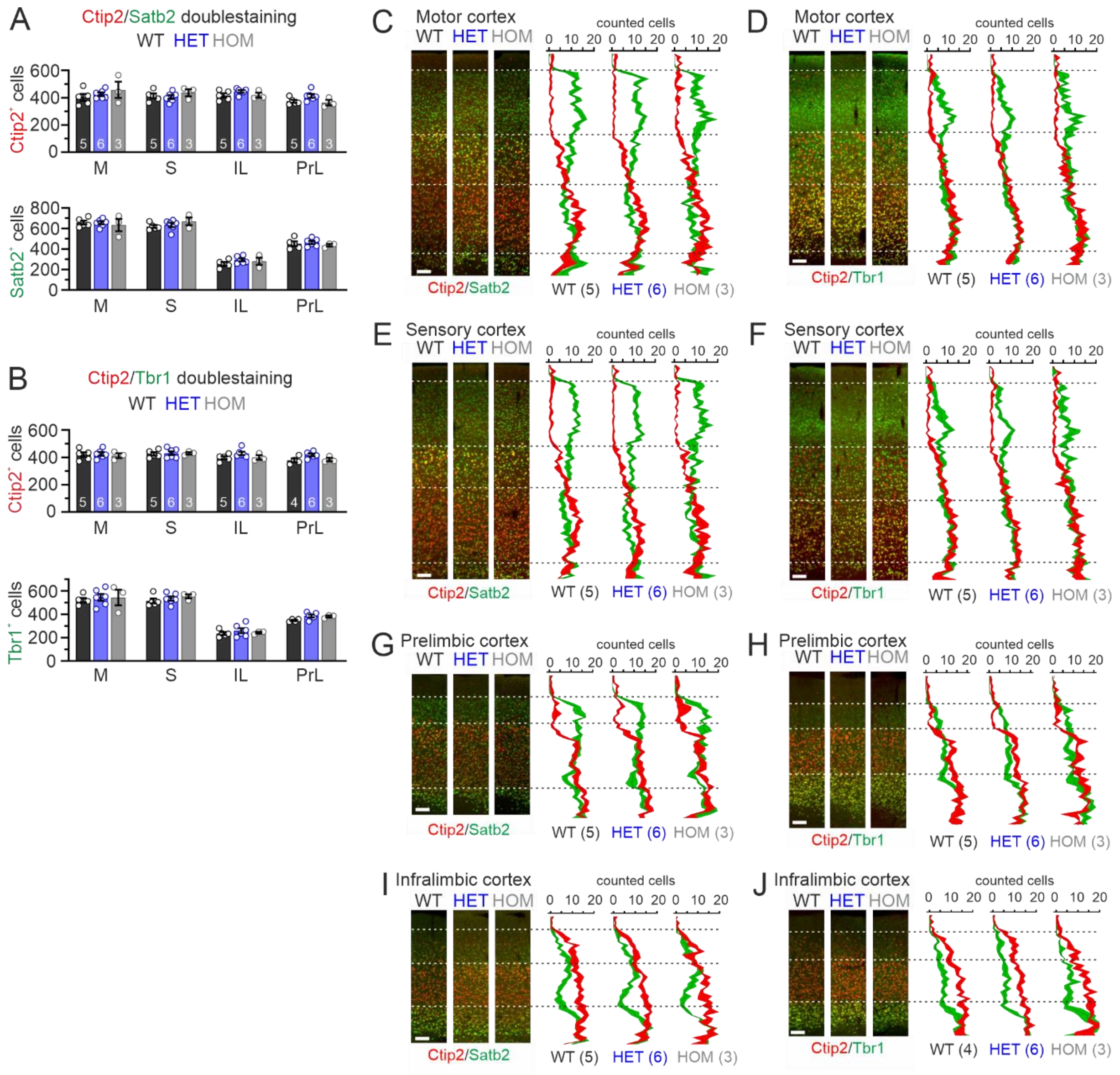

Supplemental Figure 6: Similar cortical cell number and layering in Cav1.3^{AG} mutant mice compared to WT. Two adjacent 40 µm thick coronal sections from adult Cav1.3^{AG} WT and mutant mice (~12 wk) at Bregma 1.78-1.94 (according to The Mouse Brain in Stereotaxic Coordinates Third Edition [5]) were double-stained against Ctip2/Satb2 or Ctip2/Tbr1 to visualize the layering of the following cortical regions: motor cortex (M1), sensory cortex (S1), infralimbic (IL) and prelimbic (PrL) cortex. Data are given as mean ± SEM. **A,B,** Total Ctip^{+}, Satb2^{+} or Tbr1^{+} cells within the selected region of interest (see Methods). Two-way ANOVA analysis did not reveal a statistically significant difference. **C-J,** Representative pictures of the analyzed region of interest (left) and respective population counts shown as mean ± SEM for Ctip2/Satb2 (**C,E,G,J**) or Ctip2/Tbr1 (**D,F,H,J**) stained sections for the indicated number of animals per cortical region (right). Scale bar 100 µm.

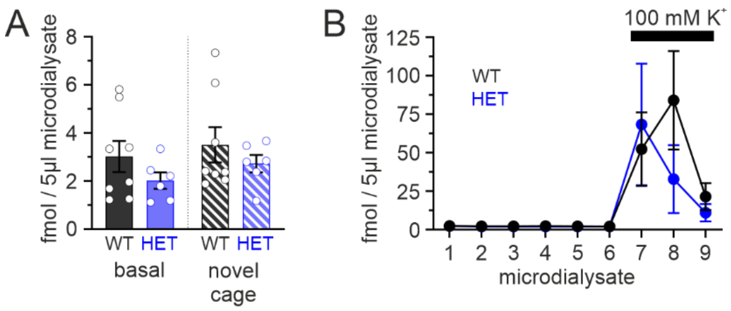

Supplemental Figure 7: Similar extracellular DA levels in the DMS of WT and HET male mice measured by in vivo microdialysis in freely moving animals. Means ± SEM. For experimental details see Supplemental Methods. **A,** Extracellular DA levels in WT (n=8) and HET animals (n=6) were similar under basal conditions, i.e. in the home cage (left), as well as after transfer of the mice into a novel cage without bedding material (handling + novel environment; right). **B,** At the end of the experiment, dialysis with high K^+ (100 mM; indicated with the black bar) resulted in a significant increase of extracellular DA in both genotypes (6 ten minutes microdialysates before perfusion with KCl containing aCSF are shown).

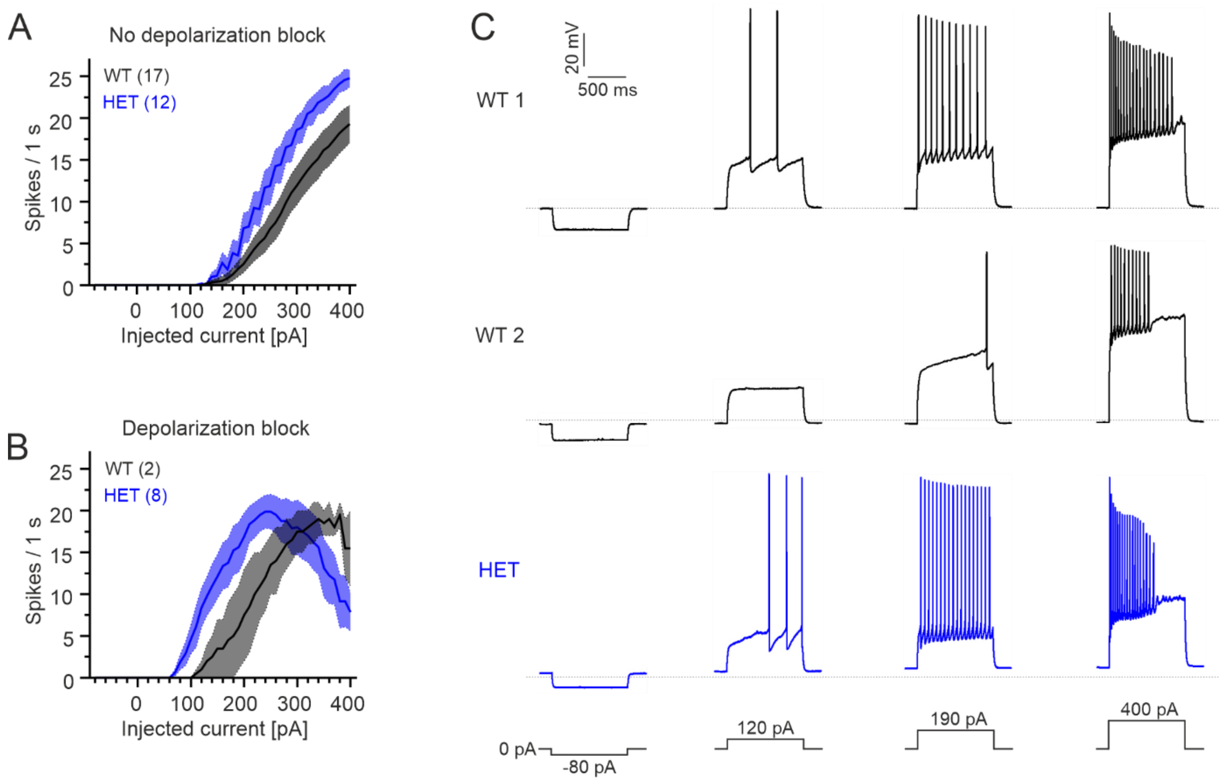

Supplemental Figure 8: Separate analysis of MSNs with or without depolarization block. A,B, Current-response curves (mean ± SEM) in response to 1 s long current injections from -80 to 400 pA of MSNs without (**A**; WT: 90%, HET: 60%) or with (**B**) depolarization block. Both behaviors were observed in independent preparation. In both populations, HET MSNs showed a hyperexcitable phenotype, evident as spike firing at lower current injections (**A+B**) or higher maximal number of fired action potential spikes (**A**). Cells with depolarization block were characterized by a higher input resistance (WT: 78.1±5.6 vs 144.0±21.0; HET: 93.4±7.0 vs 143.5±15.3, p=0.0037 unpaired Student's t-test) and a more hyperpolarized resting membrane potential (WT: -82.0±0.6 vs -81.4±1.1; HET: -80.2±1.0 vs -75.7±1.1, p=0.01 unpaired Student's t-test). Since only two WT MSNs displayed a depolarization block, the n-number was too low for statistical analysis. **C**, Representative traces of the two WT cells that displayed a depolarization block at high current injections (starting at 370 or 390 pA step; black traces at the top) and of one HET MSNs (starting from 240 to 390 pA steps, on average at 323.8±17.8 pA; blue trace).

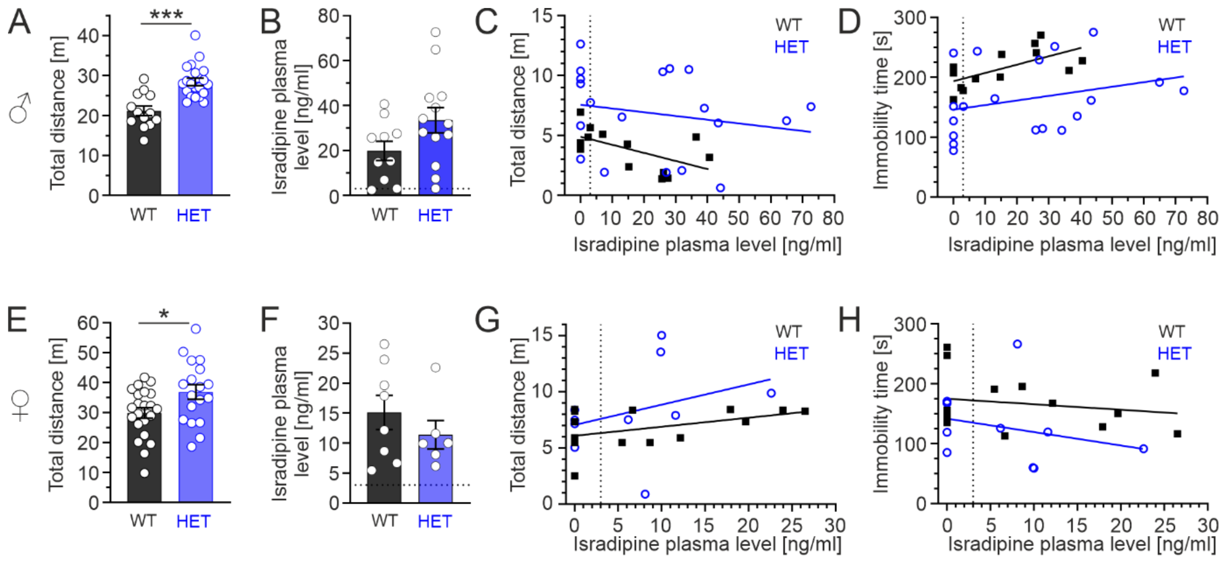

Supplemental Figure 9: Hyperlocomotion in drug naïve HET mice and correlation of isradipine plasma levels with locomotion parameters. Data for male (top; **A-D**) and female (bottom; **E-H**) WT and HET mutant mice are given as means ± SEM. **A+E**, In the drug naïve state HET mice of both sexes showed increased hyperlocomotion in the open field test, evident as enhanced total distance travelled (**A,** WT n=13, HET n=20; **E,** WT n=22, HET n=18; unpaired Student's t-test). Also, immobility time was significantly decreased in both sexes (not shown; male WT 192.8 ± 12.1, HET 107.1 ± 7.3, p<0.001, unpaired Student's t-test; female WT 135.8 ± 13.9, HET 97.0 ± 14.0, p=0.0341, Mann-Whitney test). Anxiety-related parameters did not differ between genotypes (data not shown). **B+F**, Plasma was taken immediately after the EPM (**B,** 4-5 h after the last dose; **F,** 2 h after the last dose; for details see Figure 7 and Method section) and isradipine plasma levels were measured. **B,** Male mean plasma level WT: 19.9±4.3 ng/ml (n=10); HET: 33.4±5.6 ng/ml (n=13); one HET was excluded due to levels below the limit of quantification. **F,** Female mean plasma level WT: 15.1±2.8 (n=8); HET: 11.4±2.4 (n=6). Distance travelled (**C,G**) or time spent immobile (**D,H**) plotted against the isradipine plasma level. Vertical dashed line indicates 3 ng/ml (8.1 nM), above which levels are considered therapeutically relevant in humans. **C-D**, A significant correlation was found for male WT animals (Pearson correlation; distance travelled: r^2=0.3322, p=0.0392; immobility time: r^2=0.4110, p=0.0182), while HET mice showed a trend towards reduced locomotion (Pearson correlation; distance travelled: r^2=0.04066, p=0.4078; immobility time: r^2=0.08554, p=0.2243). **G-H,** At lower isradipine plasma levels achieved in females no such correlation was observed (Pearson correlation; WT: distance r^2=0.2005, p=0.0942; immobility r^2=0.03780, p=0.4875; HET: distance r^2=0.1086, p=0.3523; immobility r^2=0.06847, p=0.4652). One WT animal was

excluded due to problems with the video and two HET animals (vehicle + isradipine group) were excluded as they jumped off the EPM.

References

1. Pinggera, A., et al., *CACNA1D De Novo Mutations in Autism Spectrum Disorders Activate Cav1.3 L-Type Calcium Channels.* Biol Psychiatry, 2015. **77**(9): p. 816-22.
2. Striessnig, J., *Voltage-Gated Ca(2+)-Channel alpha1-Subunit de novo Missense Mutations: Gain or Loss of Function - Implications for Potential Therapies.* Front Synaptic Neurosci, 2021. **13**: p. 634760.
3. Bock, G., et al., *Functional properties of a newly identified C-terminal splice variant of Cav1.3 L-type Ca2+ channels.* J Biol Chem, 2011. **286**(49): p. 42736-48.
4. Singh, A., et al., *Modulation of voltage- and Ca^{2+}-dependent gating of Cav1.3 L-type calcium channels by alternative splicing of a C-terminal regulatory domain.* J Biol Chem, 2008. **283**(30): p. 20733-44.
5. Franklin, K.B.J. and G. Paxinos, *The Mouse Brain in Stereotaxic Coordinates, 3rd Edition.* Academic Press, 2007.
